# The Pore-Forming Toxin Listeriolysin O Mediates a Novel Entry Pathway of *L. monocytogenes* into Human Hepatocytes

**DOI:** 10.1371/journal.ppat.1002356

**Published:** 2011-11-03

**Authors:** Stephen Vadia, Eusondia Arnett, Anne-Cécile Haghighat, Elisabeth M. Wilson-Kubalek, Rodney K. Tweten, Stephanie Seveau

**Affiliations:** 1 Departments of Microbiology and Internal Medicine, Center for Microbial Interface Biology, Ohio State University, Columbus, Ohio, United States of America; 2 Department of Cell Biology, The Scripps Research Institute, La Jolla, California, United States of America; 3 Department of Microbiology and Immunology, University of Oklahoma Health Sciences Center, Oklahoma City, Oklahoma, United States of America; Institut Pasteur, France

## Abstract

Intracellular pathogens have evolved diverse strategies to invade and survive within host cells. Among the most studied facultative intracellular pathogens, *Listeria monocytogenes* is known to express two invasins-InlA and InlB-that induce bacterial internalization into nonphagocytic cells. The pore-forming toxin listeriolysin O (LLO) facilitates bacterial escape from the internalization vesicle into the cytoplasm, where bacteria divide and undergo cell-to-cell spreading via actin-based motility. In the present study we demonstrate that in addition to InlA and InlB, LLO is required for efficient internalization of *L. monocytogenes* into human hepatocytes (HepG2). Surprisingly, LLO is an invasion factor sufficient to induce the internalization of noninvasive *Listeria innocua* or polystyrene beads into host cells in a dose-dependent fashion and at the concentrations produced by *L. monocytogenes*. To elucidate the mechanisms underlying LLO-induced bacterial entry, we constructed novel LLO derivatives locked at different stages of the toxin assembly on host membranes. We found that LLO-induced bacterial or bead entry only occurs upon LLO pore formation. Scanning electron and fluorescence microscopy studies show that LLO-coated beads stimulate the formation of membrane extensions that ingest the beads into an early endosomal compartment. This LLO-induced internalization pathway is dynamin-and F-actin-dependent, and clathrin-independent. Interestingly, further linking pore formation to bacteria/bead uptake, LLO induces F-actin polymerization in a tyrosine kinase-and pore-dependent fashion. In conclusion, we demonstrate for the first time that a bacterial pathogen perforates the host cell plasma membrane as a strategy to activate the endocytic machinery and gain entry into the host cell.

## Author Summary

*Listeria monocytogenes* is responsible for the severe foodborne disease listeriosis. During pathogenesis, invasion of nonphagocytic cells by *L. monocytogenes* is crucial for crossing the host epithelial barriers and colonization of multiple organs including the liver. In this study, we investigated the role of the pore-forming toxin listeriolysin O (LLO) in *L. monocytogenes* entry into human hepatocytes. LLO belongs to the largest family of bacterial pore-forming toxins called the cholesterol-dependent cytolysins and is a major virulence factor of *L. monocytogenes*. We observed that LLO is required for efficient entry of *L. monocytogenes* into hepatocytes and shed light on the molecular processes involved in this activity. Using different experimental approaches, we provide the first evidence that LLO is sufficient to induce bacterial internalization into host cells by a pore-dependent mechanism. LLO induces tyrosine kinase(s)-, dynamin-, and F-actin-dependent formation of an internalization vesicle. Similar to LLO, the pore-forming toxin pneumolysin regulates bacterial entry into host cells. Together, these findings indicate that host membrane perforation by a pore-forming toxin can be used as an invasion strategy by *L. monocytogenes* and raise the hypothesis that other bacteria may use a similar entry pathway.

## Introduction

Despite the diversity of virulence factors promoting host cell invasion, only two major mechanisms of entry have been observed [1]–[3]. First, invasins on the bacterial cell surface bind to host cell receptors to activate complex signaling cascades that orchestrate the internalization of the bacterium. Second, some bacteria bypass the requirement for a host receptor by utilizing a secretion system that injects effectors into the host cell. The effectors subvert the host signaling machinery to trigger bacterial uptake into macropinosomes [4], [5]. Using *Listeria monocytogenes* as a model intracellular pathogen, we have analyzed a novel entry pathway that is activated in response to host cell perforation by a pore-forming toxin.

*L. monocytogenes* is a foodborne pathogen that causes a large spectrum of clinical manifestations ranging from gastroenteritis to life-threatening meningo-encephalitis and sepsis. Susceptible hosts include the elderly and immunocompromised individuals [6]. In pregnant women, the bacterium can cross the maternofetal barrier causing abortion, stillbirth, and neonatal meningitis or sepsis [6]–[8]. To cross the host barriers and infect various organs including the liver [9]–[12], *L. monocytogenes* expresses multiple virulence factors that induce its entry and survival into various nonphagocytic cells [13]–[16].

Two major genetic loci encode the virulence factors responsible for host cell invasion: the internalin operon and *Listeria* pathogenicity island 1 (LIPI-1) [17], [18]. The internalin operon encodes internalin (InlA) and InlB that bind to E-cadherin and the hepatocyte growth factor receptor (HGF-Rc/c-Met), respectively [19], [20]. Depending on the receptors expressed by the host cells, *L. monocytogenes* entry involves one or both internalins [21], [22]. The internalin/host receptor interactions activate signaling cascades within cholesterol-rich microdomains leading to the internalization of the bacterium [23]–[26]. After internalization, the secreted pore-forming toxin listeriolysin O (LLO) and two phospholipases (encoded by LIPI-1) mediate *L. monocytogenes* escape from the endocytic vesicle into the cytoplasm, where bacteria divide and undergo F-actin based motility to spread from cell to cell [27]–[29].

InlA and InlB were defined as bacterial invasins based upon their critical role in *L. monocytogenes* invasion of nonphagocytic cells, and the fact that they are sufficient to induce entry of noninvasive bacteria when overexpressed from a plasmid [17], [30]. It is well established that the internalins are critical for host cell invasion; however, they may not be sufficient for inducing efficient bacterial uptake due to their low levels of expression in *L. monocytogenes*. Additional virulence factors including LLO have been proposed to regulate *L. monocytogenes* entry into host cells [31]–[35].

LLO is required for *L. monocytogenes* pathogenesis [36] and belongs to the family of the cholesterol-dependent cytolysins (CDCs) produced by numerous Gram-positive pathogens [37]–[39]. The CDCs are 50–70 kDa proteins synthesized as water soluble monomers that bind to cholesterol in host cell membranes [40]. Three members of the CDCs, intermedilysin, lectinolysin and vaginolysin, have been shown to bind to a host receptor (the complement regulatory molecule CD59) in addition to cholesterol [41]–[43]. Upon binding to host membranes, the CDCs diffuse laterally to form a ring-shaped oligomeric prepore complex. This complex then inserts a large β-barrel pore across the membrane in a cholesterol-dependent fashion [44]. Eukaryotic cells possess sophisticated mechanisms to repair damaged plasma membranes and survive moderate exposure to pore-forming toxins including the CDCs [45]. A growing body of evidence demonstrates that pores formed by pore-forming proteins including perforin, *S. aureus* alpha hemolysin, and the CDC streptolysin O (SLO), are removed from the plasma membrane through a mechanism that involves membrane internalization [45]–[47]. The ability of CDCs to induce membrane internalization in eukaryotic cells to repair their membrane raised the hypothesis that LLO may affect the internalization of *L. monocytogenes*. In the present work we explored the link between the formation of pore complexes by LLO and bacterial internalization. Using several experimental approaches, we determined that LLO is a critical invasion factor that perforates the host cell plasma membrane to activate *L. monocytogenes* internalization into human hepatocytes.

## Results

### LLO is critical for efficient entry of *L. monocytogenes* into host cells

**Figure 1:**
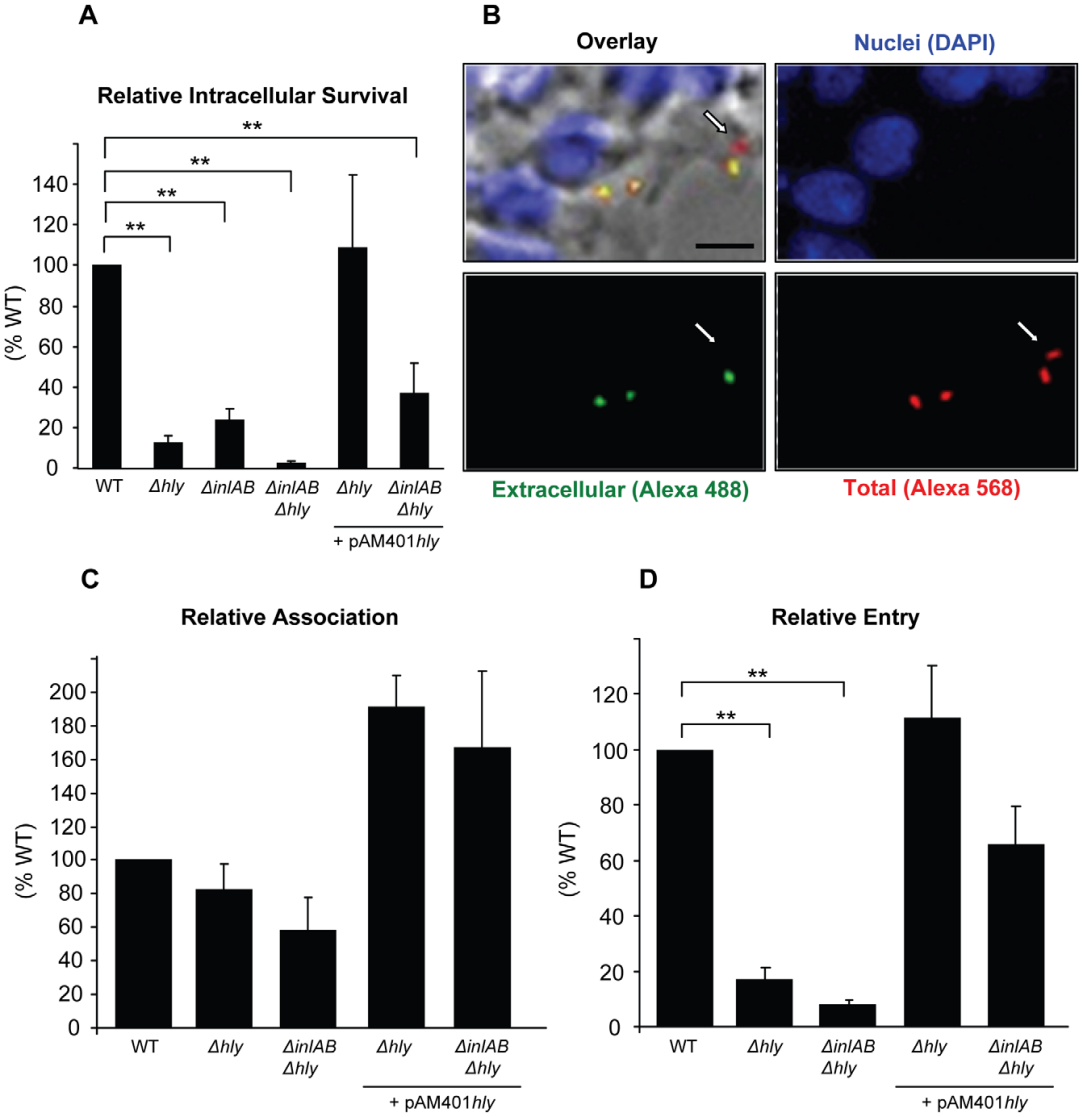
LLO is required for efficient entry of *L. monocytogenes* into HepG2 cells. (**A**) HepG2 cells were infected with isogenic WT (DP10403S), LLO-deficient (Δ*hly*), InlAB-deficient (Δ*inlAB*), LLO- and InlAB-deficient (Δ*hly*Δ*inlAB*), or LLO-complemented (Δ*hly* + pAM401*hly;* Δ*hly*Δ*inlAB +*pAM401*hly*) bacteria (MOI = 20) for 30 min at 37°C. Gentamicin was added for 1 h and the CFUs were enumerated as described in methods. Results were the mean ± SEM (n≥3) and expressed relative to WT. Statistics indicated here and elsewhere are as follows: * p<0.05; ** p<0.005. (**B**) to (**D**) Cells were infected with WT, LLO-deficient (Δ*hly*), LLO- and InlAB-deficient (Δ*hly*Δ*inlAB*), or LLO-complemented (Δ*hly* + pAM401*hly;* Δ*hly*Δ*inlAB +* pAM401*hly*) bacteria (MOI = 20) for 30 min at 37°C. Cells were washed, fixed, and labeled with fluorescent antibodies and DAPI. (**B**) Representative images of WT extracellular bacteria (green), total bacteria (red), and HepG2 nuclei (blue). The arrow indicates an internalized bacterium. Scale bar = 10 µm. In (**C**) and (**D**), results were the mean ± SEM (n≥3) and were expressed relative to WT.

The gentamicin survival assay is commonly used to assess the role of virulence factors in the intracellular survival of bacterial pathogens. This assay enumerates viable intracellular bacteria after killing extracellular bacteria with the cell impermeant antibiotic gentamicin. We measured the relative intracellular survival of wild type *L. monocytogenes* (WT), along with an isogenic LLO-deficient (Δ*hly*) mutant, a double deletion mutant deficient in the expression of InlA and InlB (Δ*inlAB*), a triple deletion mutant (Δ*hly*Δ*inlAB*), and the LLO-complemented mutant (Δ*hly* + pAM401*hly* and Δ*hly*Δ*inlAB* + pAM401*hly*) strains in HepG2 cells. As presented in Fig. 1A, efficient intracellular survival of *L. monocytogenes* required the expression of the internalins as well as LLO. In the absence of the three virulence factors, intracellular survival was almost completely abrogated. The defect in intracellular survival of LLO-deficient bacteria (Δ*hly*) was due to the lack of LLO expression, as the LLO-complemented (Δ*hly* + pAM401*hly*) and WT strains displayed similar intracellular survival.

The results obtained with the gentamicin assay reflect the efficiencies of several stages of host cell invasion including *L. monocytogenes* association with host cells, entry into host cells, escape from the internalization vesicle, and intracellular division. LLO is known to promote *L. monocytogenes* intracellular survival by mediating bacterial escape from the internalization vesicle; therefore, it was difficult to dissociate the role of LLO in escape from its potential role in bacterial association and/or entry using this assay. To specifically assess the role of LLO during the initial stages of the invasion process, we used an automated fluorescence-based assay that measures the efficiencies of bacterial association with and internalization into host cells [48]. We found that LLO and the internalins did not significantly affect bacterial association with HepG2 cells. However, LLO significantly increased bacterial association when overexpressed from a plasmid (Fig. 1C). More importantly, LLO is critical for *L. monocytogenes* internalization as we observed a marked decrease in entry of the LLO (Δ*hly*) and LLO/internalins (Δ*hly*Δ*inlAB*) deficient strains relative to the WT strain (Fig. 1D). The LLO-complemented (Δ*hly* + pAM401*hly*) and WT strains displayed similar internalization efficiencies (Fig. 1D). We also tested a second strain of *L. monocytogenes* (LO28) and its isogenic LLO-deficient mutant (LO28 *hly::Tn917*) and the human epithelial HeLa cells that are commonly used as a model to study the *L. monocytogenes* intracellular lifecycle. Again, we found that LLO is a key virulence factor that controls bacterial entry into host cells but does not significantly affect bacterial association with host cells (Fig. S1).

### Direct and dose-dependent activity of LLO in *L. monocytogenes* entry into host cells

**Figure 2:**
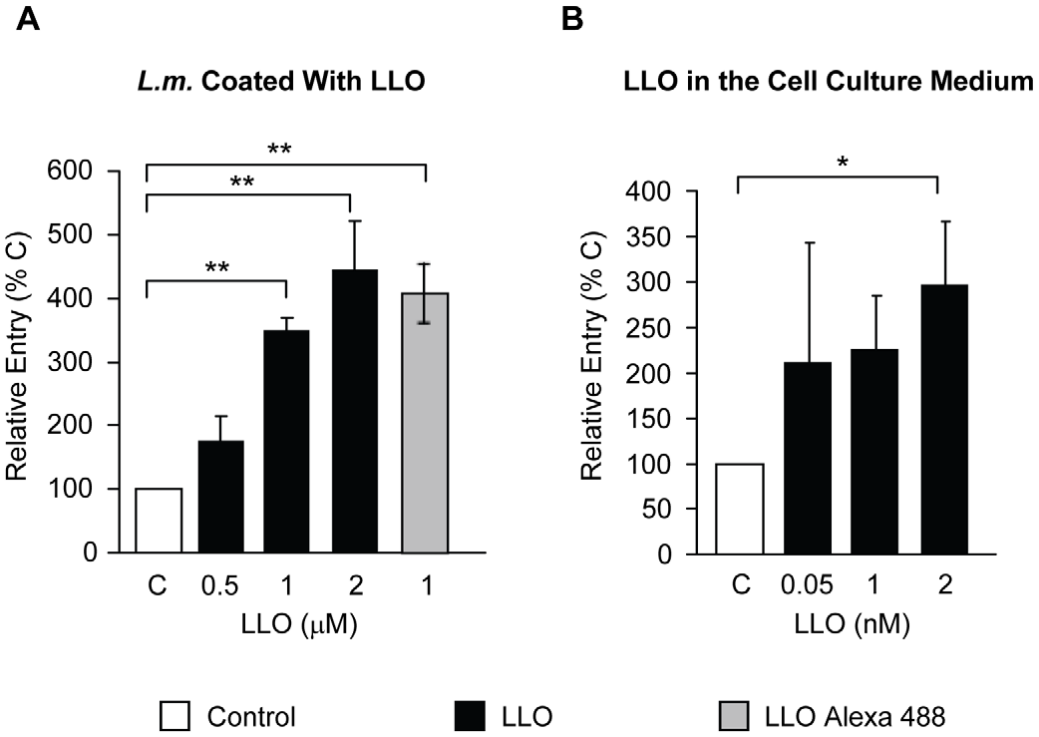
Direct and dose-dependent role of LLO in *L. monocytogenes* entry. (**A**) LLO-deficient *L. monocytogenes* (Δ*hly*, strain DPL2161, *L.m*.) treated with 1 mM nickel (II) chloride coating buffer in the absence (C) or presence of LLO (black bars) or LLO Alexa 488 (grey bar) were used to infect HepG2 cells at 37°C for 30 min (MOI = 20). (**B**) HepG2 cells were infected with LLO-deficient *L. monocytogenes* (Δ*hly*, DPL2161) at 37°C for 30 min (MOI = 20), in the absence (C) or in the presence of LLO added exogenously to the cell culture medium (black bars). In (**A**) and (**B**) bacterial entry was measured by fluorescence microscopy. The results were expressed relative to bacteria incubated with host cells in the absence of LLO (C) and were the mean ± SEM (n≥3).

The lack of LLO in the LLO-deficient strains may indirectly decrease the efficiency of bacterial entry by affecting the expression or regulation of other virulence factors. To verify that the defect in entry was solely due to the absence of LLO, we used LLO-deficient bacteria coated with increasing concentrations of six-His tagged recombinant LLO. LLO was noncovalently adsorbed on the surface of LLO-deficient *L. monocytogenes* using a previously described protocol [49]. We performed a noncovalent coating because the toxin monomers likely need to dissociate from the bacterial surface to freely diffuse within the host cell membrane and form oligomers and pores. To validate the coating procedure, we measured the fluorescence intensity of bacteria coated with increasing concentrations of an Alexa 488 LLO derivative (Fig. S2). We then measured the entry of LLO-deficient *L. monocytogenes* coated with increasing concentrations of recombinant LLO and observed that LLO increased bacterial entry into HepG2 cells in a dose-dependent fashion (Fig. 2A). We next determined whether LLO should be localized in the vicinity of the bacteria or whether LLO could act distally to regulate entry. When adding LLO to the culture medium along with the LLO-deficient strain, we observed an increase in the efficiency of bacterial internalization (Fig. 2B). Together, these data show that LLO potentiates internalization of *L. monocytogenes* into host cells in a dose-dependent fashion by acting locally or from a distance.

### LLO is sufficient to induce bacterial and bead entry into host cells

**Figure 3:**
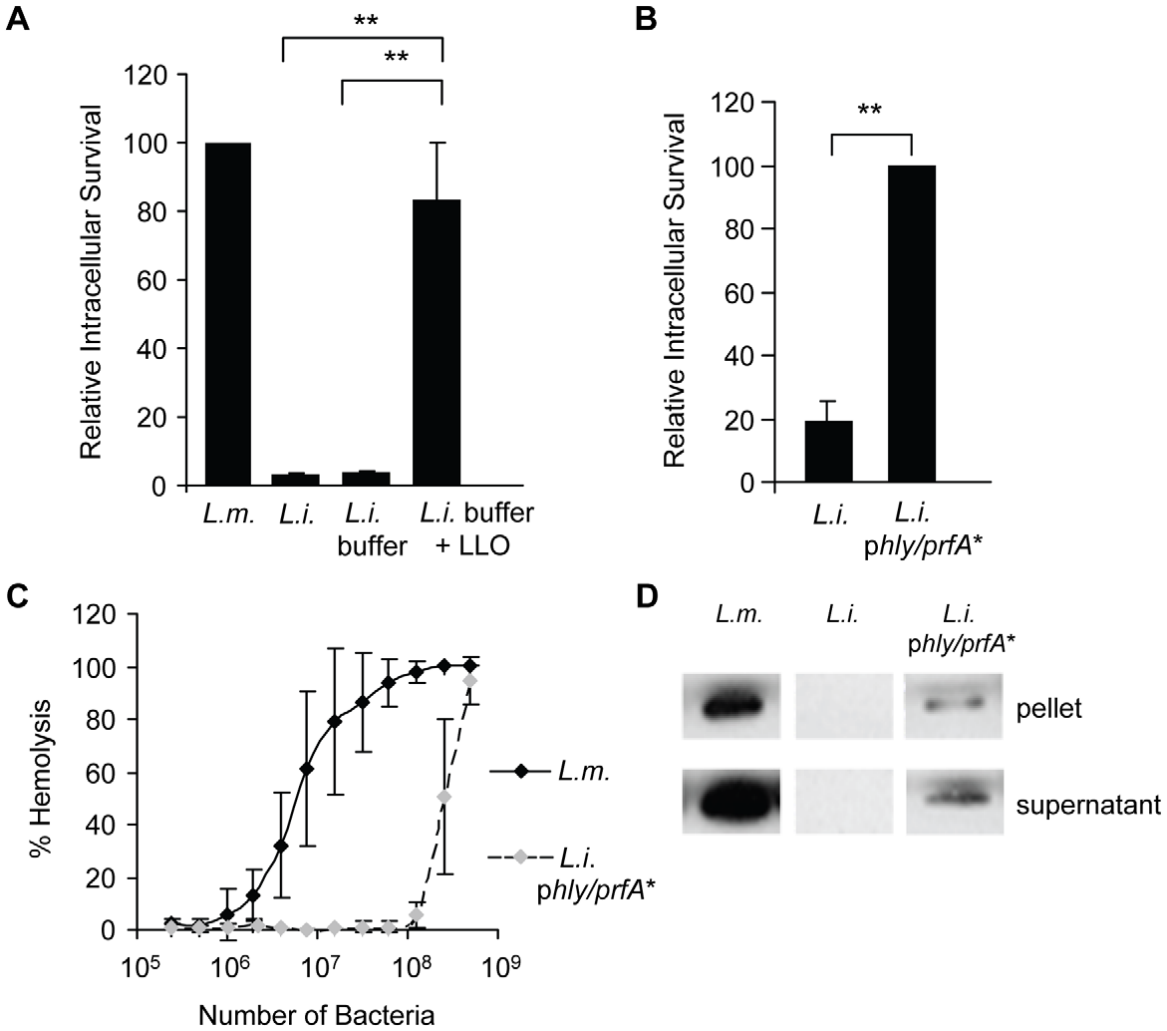
LLO is sufficient to induce the entry of noninvasive *L. innocua* into HepG2 cells. (**A**) HepG2 cells were infected with WT *L. monocytogenes* (DP10403S, *L.m.*), *L. innocua (L.i.)*, or *L. innocua* treated with 1 mM nickel (II) chloride coating buffer in the absence (*L.i.* buffer) or presence of 5 µM LLO (*L.i.* buffer + LLO) at MOI = 20 for 30 min at 37°C. Gentamicin was added for 1 h and the intracellular CFUs were enumerated. Results were the mean ± SEM (n≥3) and expressed relative to *L. monocytogenes*. (**B**) HepG2 cells were infected with *L. innocua* (*L.i.*) or *L. innocua* phly/prfA* (*L.i.* phly/prfA*) for 60 min at 37°C (MOI = 100). Gentamicin was added for 30 min and the intracellular CFUs were enumerated. Results were the mean ± SEM (n≥3) and expressed relative to *L. i.* phly/prfA*. (**C**) Hemolytic activities of *L. monocytogenes* (DP10403S) and *L. innocua* phly/prfA* measured at 37°C for 30 min, pH 7.4. Results were the mean ± SEM (n≥3). (**D**) Equivalent amounts of *L. monocytogenes* and *L. innocua* lysates and proteins precipitated from their culture supernatants were analyzed by western blotting using an anti-LLO primary antibody. A representative experiment (of 3) is presented.

**Figure 4:**
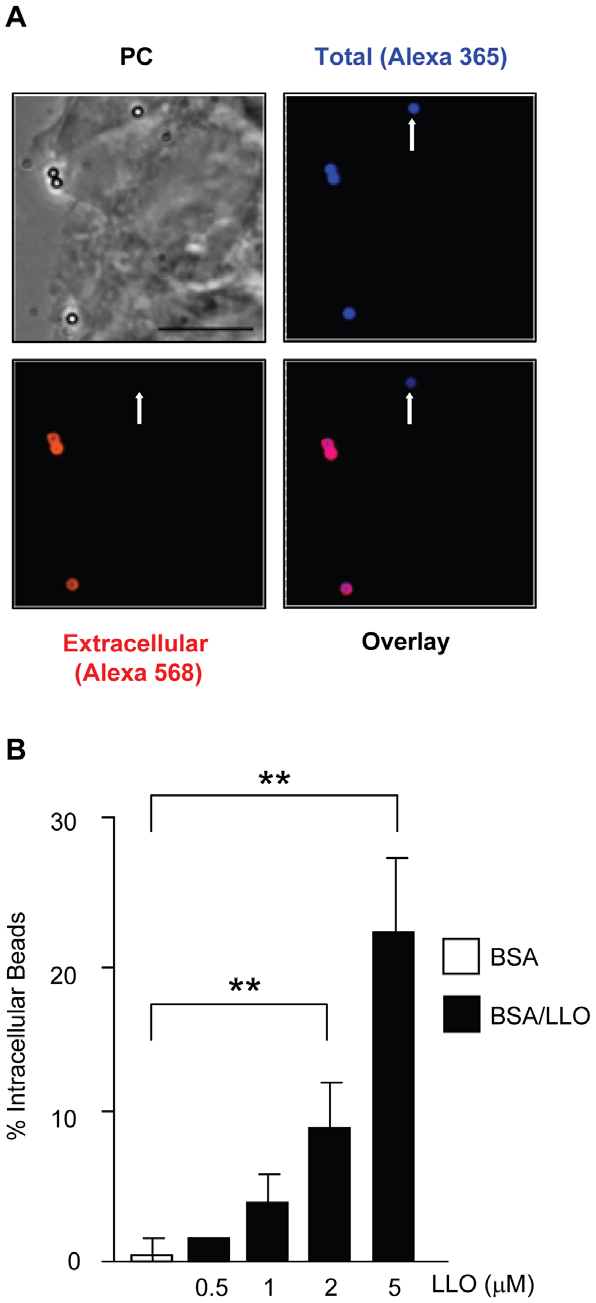
LLO is sufficient to induce entry of polystyrene beads into HepG2 cells. HepG2 cells were incubated with BSA- or BSA/LLO-coated beads for 30 min at 37°C (MOI = 20). Cells were washed, fixed, and extracellular beads were fluorescently labeled with a primary anti-BSA antibody and a secondary fluorescent antibody. (**A**) Representative fluorescence and phase-contrast (PC) images acquired with a 100 X objective. Scale bar = 10 µm. (**B**) The mean ± SEM (n≥3) percentage of intracellular beads was determined by counting a minimum of 100 beads in each sample.

We determined whether LLO is sufficient to induce bacterial entry into host cells or whether it only potentiates the internalins' activity. We used nonpathogenic and noninvasive *Listeria innocua* that does not express any of the known *L. monocytogenes* virulence factors [50]. As shown in Fig. 3A, *L*. *innocua* noncovalently coated with six-His tagged LLO were able to enter and survive in HepG2 cells. As a second approach, *L. innocua* were transformed with a plasmid coding for *hly* (*L. innocua* p*hly*/prfA*). As presented in Fig. 3B, LLO secreted from *L. innocua* p*hly*/prfA* was sufficient to induce bacterial entry and survival into HepG2 cells. The efficiency of host cell invasion by *L. innocua* p*hly*/prfA* was low compared to what was observed with WT *L. monocytogenes* and LLO-coated *L. innocua* (Fig. 3A). Because the activity of LLO in bacterial entry is dose-dependent (Fig. 2), we determined whether *L. innocua* p*hly*/prfA* expresses low levels of LLO compared to WT *L. monocytogenes*. The hemolytic activity of *L. innocua* p*hly*/prfA* was approximately 40-fold lower than *L. monocytogenes* (Fig. 3C). *L. innocua* p*hly*/prfA* also produced low levels of LLO compared with WT *L. monocytogenes* (Fig. 3D). These results demonstrate that low concentrations of LLO (below the concentration secreted by *L. monocytogenes*) are sufficient to induce bacterial entry into host cells. To assess the role of LLO in the absence of any other bacterial factor, we measured the uptake of 1 µm fluorescent polystyrene beads coated with LLO. The beads were first covalently coated with bovine serum albumin (BSA). The BSA-coated beads were then noncovalently coated with LLO [49]. LLO was sufficient to induce internalization of the beads into HepG2 cells in a dose-dependent fashion; whereas, the beads coated with only BSA were not taken up by the cells (Fig. 4A and B). This result clearly showed for the first time that a pore-forming toxin is a bacterial invasion factor. Cell viability was assessed by measuring the release of lactate dehydrogenase (LDH) and by trypan blue exclusion immediately and 24 h following toxin treatment (Fig. S3). These results show that the concentrations of LLO that lead to the optimal bead uptake by host cells do not compromise cellular integrity.

### LLO perforates host cells at physiological temperature and pH

**Figure 5:**
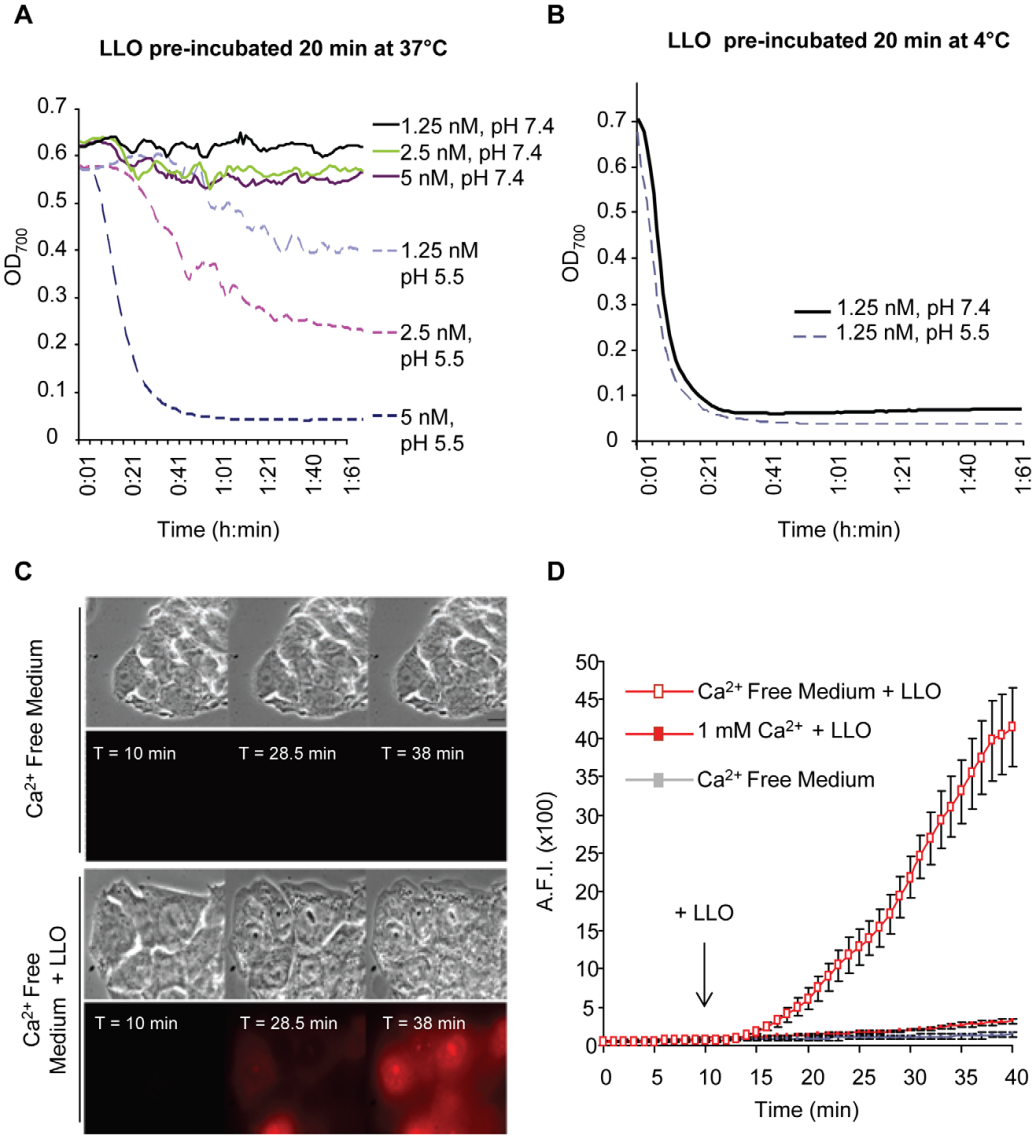
Extracellular LLO perforates erythrocytes and HepG2 cells at 37°C, pH 7.4. LLO was diluted in PBS pH 7.4 or 5.5 at 4°C in a 96 well plate (20 µl toxin in each well). (**A**) The plate was incubated at 37°C for 20 min, then, 180 µl of a 37°C suspension of sheep erythrocytes (0.25%) in PBS pH 7.4 or 5.5 was added to each well. (**B**) The plate was kept on ice for 20 min, then, 180 µl of a 37°C suspension of erythrocytes (0.25%) in PBS pH 7.4 or 5.5 was added to each well. In (**A**) and (**B**) following addition of erythrocytes, the plates were incubated at 37°C in a Power Wave 340 spectrophotometer (Bio-Tek) and absorbance (700 nm) was acquired every min [102]. Results show representative experiments (of 3), each performed in duplicate. In (**C**) and (**D**), perforation of HepG2 cells was measured by quantitative live cell fluorescence microscopy. HepG2 cells were incubated on the microscope stage at 37°C for 40 min with ethidium homodimer and in the presence or absence of 1 mM calcium. Phase contrast and fluorescence images were recorded at regular time intervals using a 100X objective and LLO was added after 10 min of incubation. Results were expressed as the averagefluorescence intensity (in arbitrary units) in the cells ± SEM of 5 movies for each condition. Scale bar = 10 µm.

Previous studies have established that pore-formation by LLO is pH sensitive at the host temperature (37°C), with LLO being more active at acidic pH. At 37°C, soluble LLO is inactivated by a pH-triggered unfurling of the domain 3 twin α-helical bundles that prevents further formation of a pore [51]. We hypothesized that the pH and temperature dependent inactivation would not occur if LLO was incubated in the presence of host membranes. Presumably, LLO would bind to the cell membrane where it would assemble into functional pores rather than unfolding in a nonproductive fashion. This hypothesis was based upon the observation that LLO is active when added to a solution of erythrocytes at 37°C and neutral pH (all of our hemolytic assays were carried out at 37°C, pH 7.4). To test this hypothesis, we pre-incubated LLO for 20 min in the absence of host cell membranes (in PBS at 37°C, pH 7.4 or 5.5) before performing the hemolytic analysis at 37°C (pH 7.4 or 5.5). Consistent with the previous observation of Schuerch et al. [51], pre-incubating the toxin at neutral pH and 37°C inactivated LLO; whereas, at acidic pH some activity of the toxin was retained (Fig. 5A). If, however LLO was pre-incubated at 4°C for 20 min before performing the hemolytic assay at 37°C, LLO retained its activity at pH 7.4 and 5.5 (Fig. 5B). These data show that soluble monomers of LLO are inactivated at 37°C and pH 7.4 in the absence of membrane, but in the presence of membranes LLO can rapidly bind to the membrane and form pores.

In response to membrane perforation by pore-forming toxins, host cells reseal their membranes using a repair process that is activated upon the influx of extracellular calcium [45]. To further demonstrate that low concentrations of LLO efficiently perforate HepG2 cells at physiological temperature and pH, we measured host cell perforation in the presence and absence of extracellular calcium. Membrane perforation was quantified by fluorescence imaging using the membrane impermeant dye ethidium homodimer. In solution this dye is weakly fluorescent, but once host cells are perforated, it enters the cells and associates with nucleic acids, which increases its fluorescence quantum yield. As shown in Fig. 5C and D, a baseline level of fluorescence was detected with host cells exposed to ethidium homodimer in calcium-free buffer at 37°C, pH 7.4 (Videos S1 and S2). LLO induces a massive entry of ethidium homodimer into cells incubated in calcium-free buffer (Videos S3 and S4), but not in the presence of 1 mM extracellular calcium. The extensive perforation of host cells was not due to the toxicity of ethidium homodimer, as in its absence LLO induces cell swelling and lysis with similar kinetics (Video S5). In total, these results showed that at 37°C, extracellular LLO efficiently perforates host cells at neutral pH.

### Construction and characterization of novel LLO variants unable to form pores

**Figure 6:**
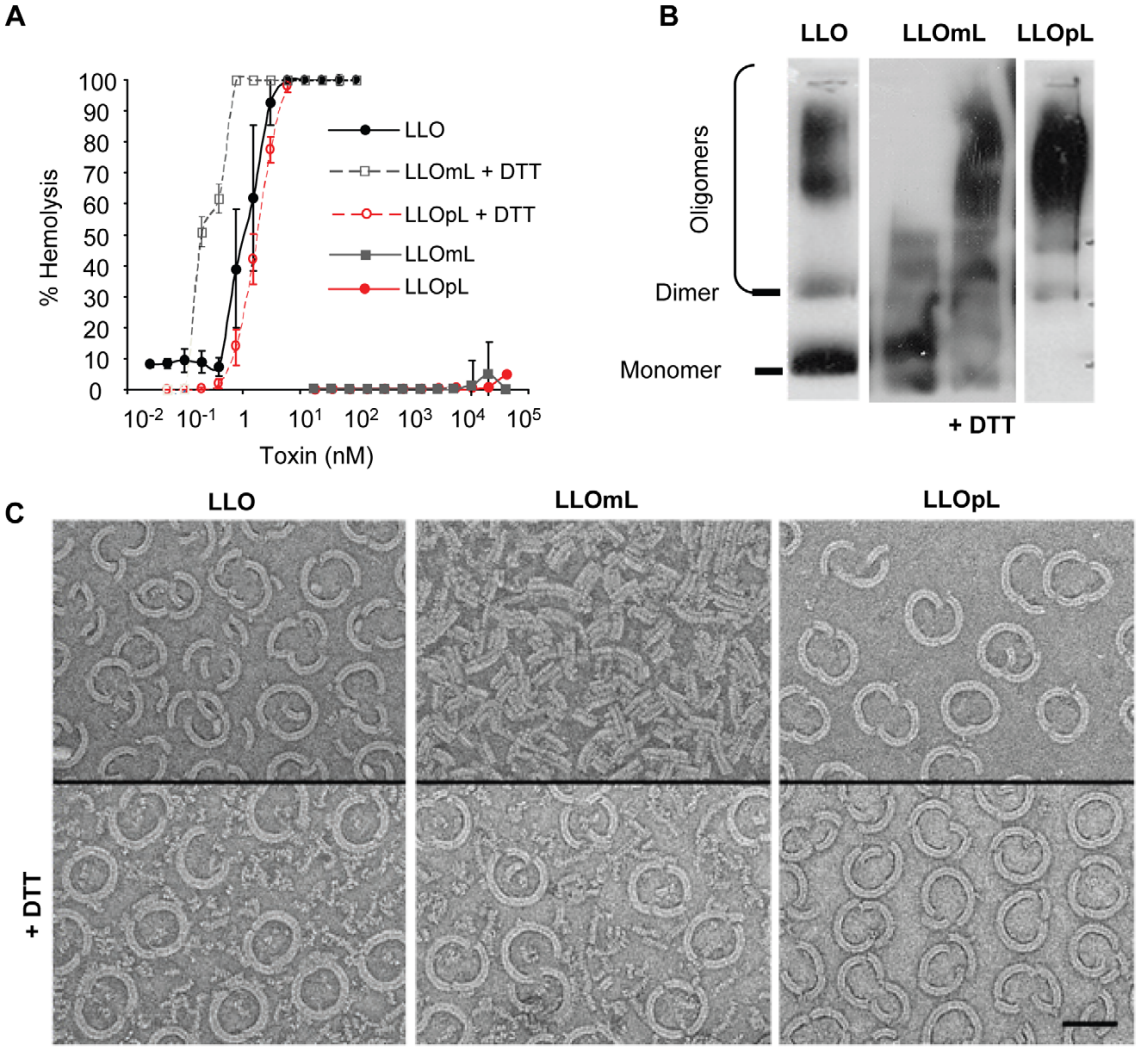
Characterization of the LLO variants. (**A**) Hemolytic activity of LLO, LLOmL, and LLOpL was measured at pH 7.4, 37°C. DTT  =  dithiothreitol. (**B**) LLO, LLOmL, and LLOpL were incubated with erythrocyte ghost membranes, in the presence or absence of DTT, washed and suspended in 1% detergent. The samples were subjected to gradient electrophoresis, in the presence of 1% detergent, and LLO was detected by western blotting using anti-LLO antibodies. (**C**) Toxins were incubated for 1 h on cholesterol/DOPC lipid layers in the presence or absence of DTT. The samples were processed and analyzed by transmission electron transmission microscopy. Scale bar = 50 nm.

To determine the importance of toxin oligomerization and pore formation in LLO-induced bacterial and bead entry into host cells, we constructed LLO derivatives locked at different stages of the pore-forming mechanism. Studies performed with the CDC perfringolysin O showed that the introduction of two cysteines at specific locations to form an intramolecular disulfide bond inhibits the hemolytic activity of the toxin [52], [53]. Depending on its location, the disulfide bond impedes the conformational remodeling required for formation of oligomers and/or pores, while toxin binding to host membranes is unchanged [52]. Based on these studies, we constructed and characterized LLOmL (monomer-locked) and LLOpL (prepore-locked). LLOmL was expected to bind to host membranes as a monomer unable to rearrange into a prepore complex. LLOpL was expected to bind to host membranes and oligomerize to form a stable prepore complex that cannot undergo the final transition into a pore. Neither mutant exhibited detectable hemolytic activity up to concentrations of 50 µM (higher concentrations were not tested). The loss of activity was due to the formation of the disulfide bonds, as reduction by dithiothreitol (DTT) fully restored the native hemolytic activity of the toxins (Fig. 6A). We next determined the oligomerization state of the toxins associated with erythrocyte membranes. As expected, LLO and LLOpL formed detergent-resistant high molecular weight complexes, whereas LLOmL failed to form such oligomers (Fig. 6B). Importantly, the addition of DTT unlocked LLOmL, as shown by the formation of detergent-resistant high molecular weight oligomers. Finally, the arrangement of the toxins associated with cholesterol-rich lipid layers was analyzed by transmission electron microscopy (Fig. 6C). LLO and LLOpL formed characteristic arc- and ring-shaped oligomers with a diameter of ∼50 nm in the presence and absence of DTT. LLOmL formed short linear assemblies that may be representative of the early stages of toxin oligomerization before formation of the prepore complex. Importantly, LLOmL formed the typical arc- and ring-shaped oligomers when it was reduced by DTT.

### LLO-induced bacterial and bead entry requires host cell membrane perforation

**Figure 7:**
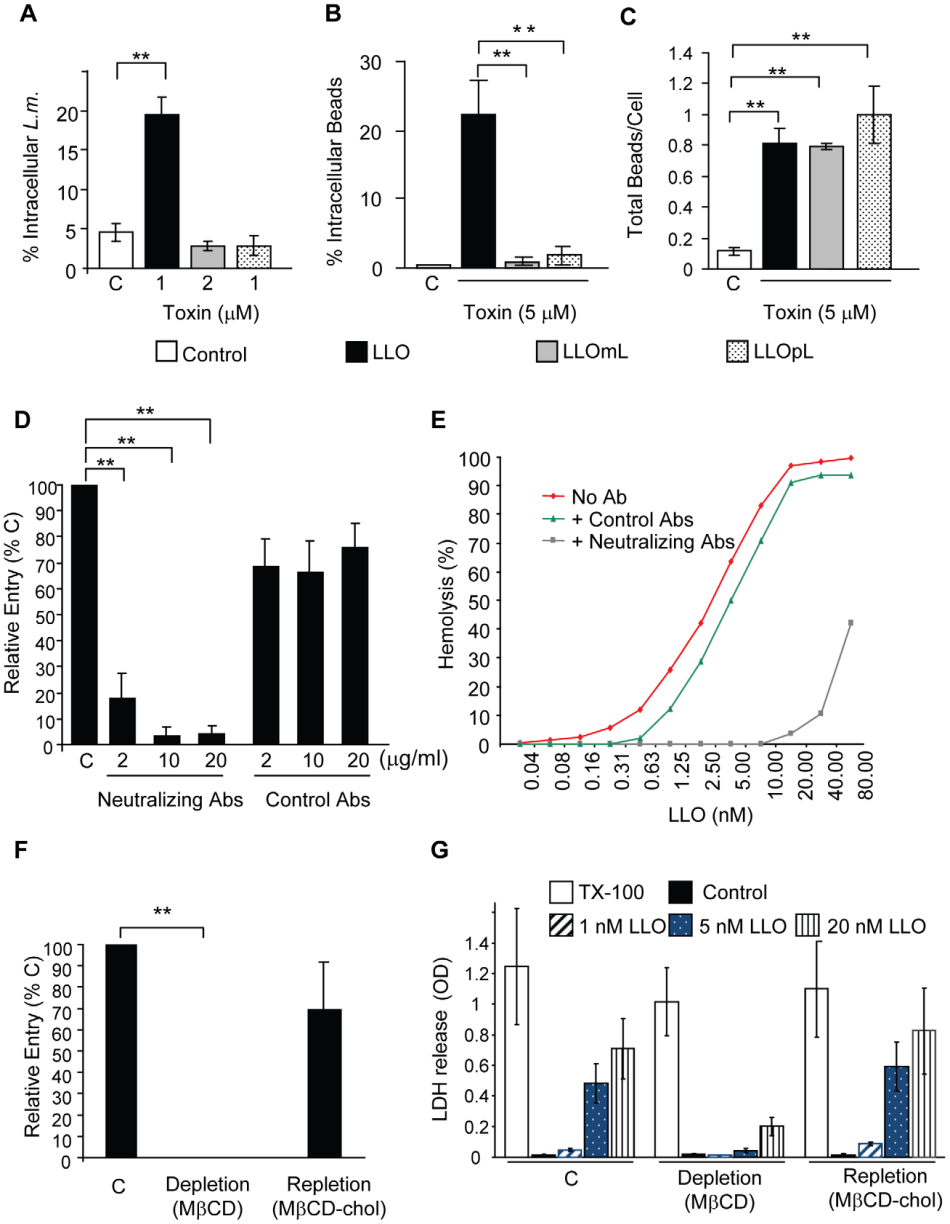
Formation of pore complexes is required for efficient bacterial and bead entry into HepG2 cells. (**A**) LLO-deficient *L. monocytogenes* (Δ*hly, L.m.*) were treated with coating buffer in the absence (white bars, C), or presence of LLO, LLOmL, or LLOpL. Cells were infected at 37°C for 30 min (MOI = 20). Samples were washed, fixed, and fluorescently labeled to enumerate bacterial entry by fluorescence microscopy. Results were the mean ± SEM (n≥3). (**B**) and (**C**) HepG2 cells were incubated with BSA- (Control; C) or BSA/toxin (LLO, LLOmL, or LLOpL)-coated beads for 30 min at 37°C (MOI = 20). Cells were washed, fixed, and labeled to enumerate bead entry (**B**) and association (**C**) by fluorescence microscopy. Results were the mean ± SEM (n≥3). (**D**) HepG2 cells were incubated with BSA/LLO-coated beads for 30 min at 37°C, in the presence or absence (C) of LLO-neutralizing or control antibodies. Cells were washed, fixed, and fluorescently labeled. Bead entry was measured by fluorescence microscopy and the results, mean ± SEM (n≥3) were expressed relative to the control (C). (**E**) Representative LLO hemolytic curves in the presence or absence (No Ab) of the neutralizing or control antibodies (10 µg/ml) were performed at pH 7.4, 37°C. (**F**) Control (C), cholesterol-depleted and -repleted HepG2 cells were incubated with BSA/LLO-coated beads for 30 min at 37°C. Bead entry was measured by fluorescence microscopy and the results, mean ± SEM (n≥3) were expressed relative to the control. (**G**) Control (C), cholesterol-depleted and -repleted HepG2 cells were incubated with various concentrations of LLO, 0.2% TX-100, or MEM (Control) at 37°C for 30 min. LDH release was measured using the TOX7 assay kit. Results are the mean ± SEM (n≥3).

We determined the role of LLO oligomerization into prepore and pore complexes in bacterial and bead entry into HepG2 cells. Native LLO induced bacterial and bead internalization, whereas neither LLOmL- nor LLOpL-coated beads were taken up by the cells (Fig.7A and B) even though all three bind to host cells with similar efficiency (Fig. 7C). We also used anti-LLO neutralizing antibodies, which were previously shown to prevent formation of LLO pores [54], to block LLO-mediated uptake of beads. The LLO neutralizing antibodies markedly inhibited LLO hemolytic activity and bead internalization, whereas control anti-LLO antibodies did not (Fig. 7D and E). The prepore to pore conversion of the CDCs is known to require high concentrations of membrane cholesterol [55]. We therefore prevented the formation of membrane pores by depleting host cholesterol using the cholesterol chelating agent methyl β-cyclodextrin (MβCD). Cholesterol depletion completely abrogated LLO-induced host cell perforation and the entry of BSA/LLO-coated beads into HepG2 cells (Fig. 7F and G). To demonstrate that inhibition of bead entry and host cell perforation was specifically due to cholesterol depletion and not to a secondary effect of the MβCD, we show that cholesterol repletion restored bead uptake and membrane perforation by LLO (Fig. 7F and G).

We also observed that BSA/LLO-coated beads formed pores in host cell membranes as detected by propidium iodide incorporation (Fig. S4A and B). Membrane perforation is a key event in LLO-induced entry. Therefore, we hypothesized that a heterologous CDC should also increase *L. monocytogenes* internalization. LLO-deficient *L. monocytogenes* were coated with recombinant six-His tagged pneumolysin (PLY), the CDC produced by *Streptococcus pneumoniae* [56] that exhibited a similar hemolytic activity to LLO (Fig. S5A). Like LLO, PLY was able to mediate *L. monocytogenes* entry into HepG2 cells (Fig. S5B). These data suggested that the LLO structure did not contain unique features that were required to induce bacterial invasion.

### LLO activates a clathrin-independent, but dynamin-dependent internalization pathway

**Figure 8:**
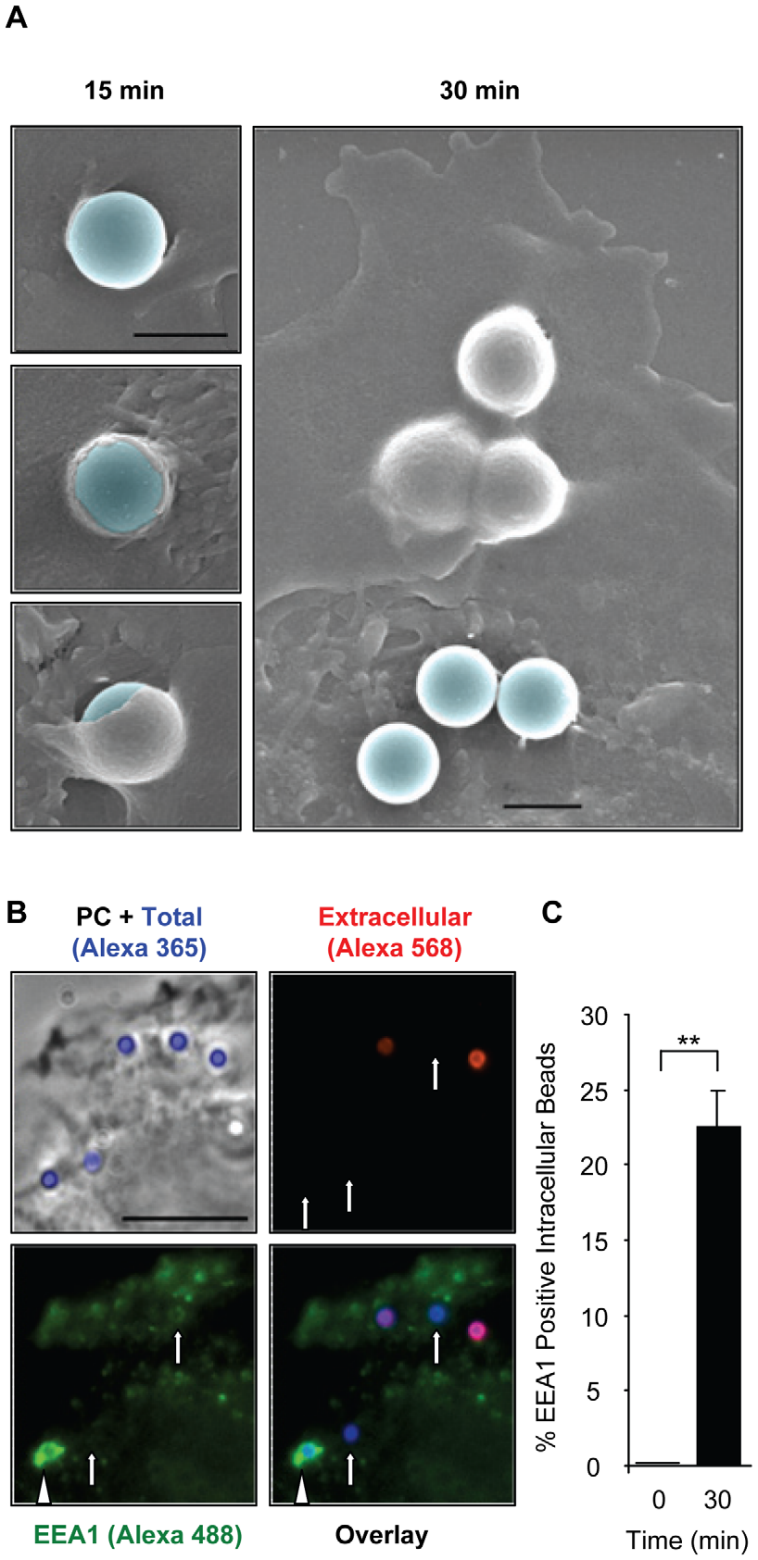
LLO-coated beads are internalized into EEA1 positive endosomes. (**A**) HepG2 cells were incubated at 37°C with BSA/LLO-coated beads for 15 or 30 min, washed, fixed, and processed for scanning electron microscopy analysis. Scale bar = 1 µm. (**B**) HepG2 cells were incubated for 30 min at 37°C with BSA/LLO-coated beads, washed, fixed, and the extracellular beads were labeled with anti-BSA antibodies and a fluorescent secondary antibody (red). After permeabilization, EEA1 was labeled using anti-EEA1 antibodies and a fluorescent secondary antibody (green). Scale bar = 10 µm. PC  =  phase contrast. Arrows point out internalized beads and the arrowhead an internalized bead that massively recruited EEA1. (**C**) Results were expressed as the mean ± SEM (n≥3) percentage of intracellular beads that recruited EEA1.

**Figure 9:**
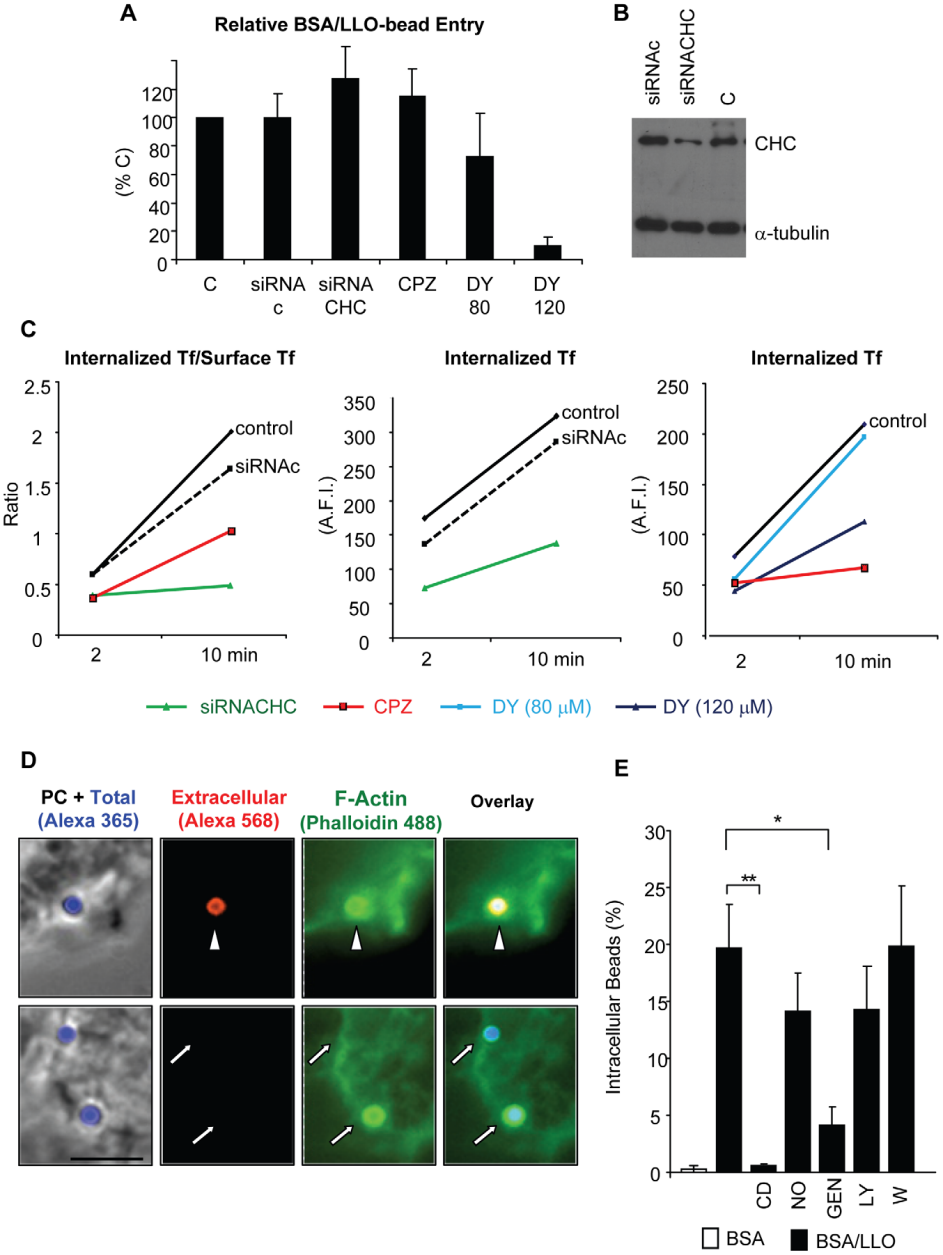
LLO-coated beads are internalized by a clathrin-independent, dynamin-, F-actin- and tyrosine kinase-dependent pathway. (A) to (C) Clathrin heavy chain was knocked down in HepG2 cells by siRNA (siRNACHC) treatment or was inhibited by pre-incubating the cells for 30 min with 10 µM chlorpromazine (CPZ). Dynamin was inhibited by pre-incubating the cells for 30 min with 120 µM dynasore (DY). CPZ and DY were maintained in the cell culture medium throughout the experiments. (A) HepG2 cells were incubated with BSA/LLO-coated beads for 30 min at 37°C. Cells were washed, fixed, and bead entry was enumerated. Results were the mean ± SEM (n≥3) and were expressed relative to the control (C). (B) Representative western blot analysis of clathrin heavy chain (CHC) in cells treated with scrambled siRNA (siRNAc), specific siRNA (siRNACHC), or control untreated cells (C). The α-tubulin was used as a loading control. (C) Measurement of internalized transferrin (Tf)/Surface associated (Tf) and internalized (Tf) in control untreated cells (C), cells treated with siRNAc or siRNACHC, and cells treated with CPZ or DY. Only internalized (Tf) was measured in DY-treated cells due to DY fluorescence. Representative experiments (of 3) are shown. (D) HepG2 cells were incubated for 30 min with BSA/LLO-coated beads, fixed, and extracellular beads were labeled using anti-BSA antibodies and secondary fluorescent antibodies (red) and F-actin was labeled with fluorescent phalloidin (green). Scale bar = 5 µm, PC  =  phase contrast. The arrowhead and arrows point out extracellular and internalized beads, respectively. (E) HepG2 cells were pre-incubated in the presence or absence of cytochalasin D (CD), nocodazole (NO), genistein (GEN), LY294002 (LY), or wortmannin (W) at 37°C. Beads were added for 30 min in the presence of the inhibitors. The percentage of internalized beads was enumerated by fluorescence microscopy and the results were the mean ± SEM (n≥3).

To demonstrate that host cell perforation by LLO leads to the formation of a micron sized internalization vesicle, we analyzed plasma membrane remodeling at the bead entry site. Scanning electron microscopy images showed the formation of plasma membrane extensions entrapping the BSA/LLO-coated beads after 15 min at 37°C. Within 30 min, we observed an increase in the number of beads completely enveloped by the plasma membrane (Fig. 8A), whereas, the formation of membrane extensions was not induced by the BSA-coated beads. To demonstrate that LLO induces entry of BSA/LLO-coated beads within a membrane-bound internalization vesicle, we quantified intracellular beads that colocalize with the early endosomal marker EEA1. After 30 min at 37°C, we observed that a large population of intracellular beads was localized in endosomes to which the EEA1 marker had been recruited (Figs. 8B and C). We further characterized the endocytic molecules involved in this pathway. We measured the entry of BSA/LLO-coated beads in cells transfected with clathrin heavy chain silencing RNA and in cells treated with the clathrin inhibitor chlorpromazine, or with dynasore, a dynamin inhibitor (Fig. 9A and B). The uptake of fluorescent transferrin was measured as a control for the inhibition of clathrin- and dynamin-dependent endocytosis (Fig. 9C). The results show that internalization of BSA/LLO-beads is dynamin-dependent and clathrin-independent.

### F-actin remodeling is induced by LLO in a pore-dependent fashion and is required for bead internalization

Internalization of large particles such as bacteria generally requires the rearrangement of subcortical F-actin to form membrane extensions that engulf the particles [57]. Consistent with this idea, the membrane rearrangements observed by scanning electron microscopy (Fig. 8A) were accompanied by the recruitment of F-actin at the bead entry site (Fig. 9D). Furthermore, bead entry was inhibited in cells treated with the F-actin depolymerizing drug cytochalasin D; whereas, microtubule integrity was not required for entry (Fig. 9E). F-actin remodeling involves the activation of the host signaling machinery. As a first approach to determine the transducers involved in entry, we have treated cells with inhibitors of tyrosine kinases (genistein) and phosphoinositide 3-kinases (PI3Ks) (LY294002 and wortmannin). Tyrosine kinases and PI3Ks are key transducers activated upstream from actin polymerization at the *L. monocytogenes* entry site [13]. We found that only tyrosine kinase(s) activation was critical for LLO-dependent entry (Fig. 9E). We also observed that purified LLO induces membrane ruffling in the 0.5 to 2 nM concentration range (representative Videos S6 and S7). Membrane ruffling started 117±14.3 sec after the addition of 1.2 nM LLO and was optimal for 555±145 sec (calculated from 10 movies). This provided a convenient experimental model to determine whether pore formation and tyrosine kinases were involved in F-actin polymerization induced by LLO. LLO-induced membrane ruffling was F-actin- and tyrosine kinase-dependent as no ruffling was observed in cells stimulated by LLO in the presence of 0.5 µg/ml cytochalasin D or 250 µM genistein (Videos S8 and S9). Importantly, membrane ruffling was only induced by LLO, but not by 0.5 to 50 nM LLOpL (Video S10). These data provide a link between membrane perforation by LLO and the remodeling of the F-actin cytoskeleton.

## Discussion

These studies revealed the existence of a novel pathway exploited by *L. monocytogenes* to gain entry into host cells. This pathway is activated in response to membrane perforation by the pore-forming toxin listeriolysin O (LLO). This is the first demonstration that a pore-forming toxin is able to induce the internalization of a bacterial pathogen into host cells. We have used several approaches to show that LLO is crucial for efficient entry of *L. monocyogenes* into HepG2 and HeLa cells and have used beads coated with LLO to specifically decipher the molecular machinery underlying this novel pathway.

LLO is known to mediate *L. monocytogenes* escape from the endocytic vesicle following bacterial internalization into host cells [27]. The present findings demonstrate that LLO is also critical for *L. monocytogenes* internalization. Moreover, they show that LLO is sufficient to induce bacterial entry. A role for LLO in bacterial entry into nonphagocytic cells was previously proposed [35], whereas other studies did not identify such a role for LLO [58]. All of these studies relied upon the gentamicin survival assay that measures intracellular survival. The gentamicin assay is a powerful method, but it exhibits some limitations. First, it reports several stages of the host cell invasion process and cannot distinguish the role of LLO in bacterial entry from its role in intracellular survival. Second, LLO perforates host cells and likely allows gentamicin entry into the cells. As a result, the intracellular survival of WT *L. monocytogenes* is underestimated in comparison to a LLO-deficient mutant. In the gentamicin assay performed in the present study, a low MOI (20) was used as higher amounts of bacteria led to substantial entry of gentamicin into the cells (MW 480), as reported by the incorporation of the cell impermeant dye ethidium homodimer (MW 857) (our unpublished data). Therefore, we developed an automated fluorescence-based assay to specifically and accurately measure bacterial association and entry [48]. With this approach, we demonstrate that LLO plays a critical role in bacterial entry, but not in bacterial association with host cells. Similar to LLO, we found that InlA and InlB did not significantly affect bacterial association, but affected bacterial intracellular survival and entry. This result is not surprising due to the abundance of adhesins expressed by *L. monocytogenes,* as over ten surface adhesins have been identified [31], [59]–[65]. When overexpressed, LLO significantly increased *L. monocytogenes* association with host cells. This result is in accordance with the observation that LLO promotes *Bacillus subtilis* attachment to epithelial cells [66]. LLO and other CDCs were also shown to remain partially bound to the bacterial cell wall [67], [68]. Therefore, when overexpressed, the cell wall-associated toxin likely anchors the bacteria to host cells via binding to membrane cholesterol. Indeed, LLO is well known to bind to cholesterol in biological and artificial membranes [40], [69].

Our study focused on elucidating the role of LLO in bacterial entry into host cells. We first investigated whether host cell perforation by LLO was required for activating this entry pathway. Although pore-formation by LLO is pH-sensitive at 37°C, we demonstrated that extracellular LLO perforates host cells at neutral pH. This finding is supported by a previous study that also concluded that LLO is active at neutral and slightly basic pH values [69]. We constructed LLO variants to determine if LLO binding to host membranes, its oligomerization into a prepore complex, and pore formation are required for LLO-induced bacterial entry. The LLO variant unable to undergo the prepore to pore transition demonstrated that the formation of LLO pore complexes is a key event for bacterial and bead internalization into host cells. How does pore-formation by LLO stimulate bacterial or bead uptake? The LLO entry pathway appears to be distinct from the canonical bacterial entry pathways. Bacteria are known to induce their entry by activating host receptors or by injecting effectors into the host cell cytosol [1], [2], [70]. Gram-positive bacteria do not have a type III secretion system, although, they can produce CDC toxins to mediate the translocation of virulence factors [71], [72]. The observation that LLO alone was sufficient to induce bacterial or bead entry ruled out this mechanism. The requirement for membrane perforation does not favor the simple model in which LLO acts by activating a signaling host receptor. Indeed, the LLOpL variant that binds to host cells and is able to rearrange into a prepore complex failed to induce bacterial entry. Also, another pore-forming toxin, PLY, could replace LLO in that function. We do not rule out the existence of a yet unknown receptor for LLO that would be shared by PLY, nevertheless, membrane perforation remains a key trigger for entry.

LLO induces the formation of internalization vesicles that accommodate large particles (bacteria or 1 µm beads) via a cholesterol-, dynamin-, and F-actin-dependent, but clathrin-independent pathway. Several clathrin-independent and dynamin-dependent internalization pathways have been described including lipid raft-dependent pathways [73]. It is important to note that the role of cholesterol in this pathway might be complex, as cholesterol is a structural component of lipid rafts and is critical for LLO binding to host membranes and the formation of LLO pores. However, the observation that LLO associates with and induces the coalescence of lipid raft microdomains favors the hypothesis of a lipid raft-mediated pathway [74]. We found that LLO induces the polymerization of actin at the bead entry site, and that F-actin dynamics and tyrosine kinase activation are required for LLO-mediated bead entry. Using soluble LLO and LLOpL, we observed that membrane perforation by LLO induces F-actin-dependent membrane ruffling. Interestingly, F-actin polymerization within membrane ruffles was tyrosine kinase-dependent. Together, our findings support a model in which host cell perforation by LLO leads to an internalization pathway that involves host tyrosine kinase-dependent stimulation of the actin cytoskeleton and the activity of dynamin. Similar to LLO, PLY promoted bacterial entry into host cells and was shown to induce actin polymerization in neuroblastoma cells [75].

The fact that LLO induces internalization of bacteria in a pore-dependent fashion is reminiscent of the membrane repair pathway observed in eukaryotic cells exposed to pore-forming toxins. In response to the attack by pore-forming proteins, eukaryotic cells undergo membrane endocytosis to remove the pores from their plasma membranes [45], [47]. We identified host cell effectors, F-actin and dynamin, that are required for LLO-induced particle internalization but are dispensable for membrane repair (Fig. S6) [45]. In conclusion, membrane repair is likely a prerequisite for LLO-induced bacteria/bead entry, but the LLO-mediated entry pathway extends beyond or is distinct from the membrane repair pathway.

The mechanisms evolved by *L. monocytogenes* to gain entry into nonphagocytic cells are complex and involve several invasins such as InlA, InlB, and LLO. The *inlA*, *inlB*, and *hly* genes are controlled by the central regulator of virulence genes, PrfA, and are highly up-regulated *in vitro* and *in vivo* during *L. monocytogenes* infection [76], [77]. Therefore, LLO is expressed together with InlA and InlB to mediate host cell invasion. Depending on the receptors expressed by host cells, InlA and InlB stimulate bacterial entry individually or in concert [13], [21]. LLO likely affects bacterial internalization in a large panel of cells because its major host receptor is cholesterol. We used hepatocytes (HepG2 cells) that express the InlA and InlB receptors, E-cadherin and the HGF-Rc, respectively [78], [79]. We also used HeLa cells that do not express E-cadherin and are only permissive to the InlB entry pathway [30]. Our results showed that LLO played a critical role in *L. monocytogenes* internalization into both cell lines. Therefore, LLO cooperation with InlB alone or with InlA and InlB is critical for bacterial internalization. Our study on LLO and previous studies on InlA and InlB showed that these molecules are individually sufficient to induce bacterial entry with high efficiency when overexpressed from a plasmid or coated on beads [17], [20], [80]. However, the expression level of these molecules is low in *L. monocytogenes* and their concerted activity likely ensures efficient bacterial uptake by host cells. The literature shows that most stages in the infectious lifecycle of a pathogenic bacterium involve numerous factors, all working in concert [21], [81], [82]. A recent study demonstrated the importance of the cooperation between InlA and InlB during *L. monocytogenes* entry into host cells [21]. In this study it was shown that InlA ensured the specificity of bacterial recognition of intestinal cells, whereas InlB increased the InlA internalization rate. Likewise, we speculate that cooperation between LLO, InlA, and InlB occurs during host cell invasion. In this model, the three pathways would contribute simultaneously to the uptake of a given bacteria. Given the identified roles of LLO, InlA, and InlB in stimulating actin polymerization and endocytosis, cooperation between the three invasins likely involves both of these processes. The LLO-dependent entry pathway displays differences and similarities with the InlA and InlB pathways. They differ with respect to the mechanism used to activate the host cells, as membrane perforation is required in the LLO but not in the InlA and InlB pathways. Also, clathrin is involved in the InlA and InlB pathways [83], but not in the LLO pathway. However, the internalization induced by the three invasins shares common effectors such as host tyrosine kinases, dynamin, cholesterol, and F-actin [13]. Determining how host cells integrate the signals simultaneously generated by each invasin and whether the resulting pathway is the sum of the individual pathways or is a new pathway leading to efficient bacterial uptake constitutes an important goal for future research.

Pore-forming toxins are produced by numerous pathogens and may influence their uptake by host cells [84], [85]. In favor of this hypothesis, while this work was under revision, it has been published that the parasite *Trypanosoma cruzi* invades host cells by exploiting a host cell membrane repair mechanism in response to membrane damage [86]. Among CDC-producing bacteria, the genera *Listeria*, *Arcanobacterium*, *Bacillus*, and *Streptococcus* include several pathogenic bacteria that have been shown to invade nonphagocytic cells [87]–[92]. Interestingly, the CDC intermedilysin (ILY) is required for internalization of *Streptococcus intermedius* [88]. However, not all of the CDCs share this property, as a recent study showed that streptolysin O (SLO) inhibits internalization of Group A *Streptococcus* into keratinocytes [93]. Therefore, a fascinating avenue of research is to elucidate the molecular mechanisms underlying the role of pore-forming toxins in the regulation of host cell invasion by intracellular pathogens.

## Materials and Methods

### Bacterial strains and plasmids

WT *L. monocytogenes* (DP10403S), isogenic Δ*hly* (*hly* is the gene coding for LLO) (DPL2161), and Δ*inlAB* (DPL4404) deletion mutants were gifts from Dr. Dan Portnoy (U.C. Berkeley, California, USA) [94]–[96]. To construct the triple deletion mutant (Δ*hly* Δ*inlAB*) we deleted *hly* in the DPL4404 strain by allelic exchange using the pKSV7 integrational shuttle vector (a gift from Dr. Nancy Freitag, University of Illinois at Chicago, USA) [97]. A ∼1000-bp DNA fragment consisting of the upstream (from bp 962 to 1463) and downstream (from bp 3029 to 3529) sequences flanking the *hly* open reading frame was amplified from *L. monocytogenes* chromosomal DNA [96]. The primers (Table S1) were designed to generate restriction sites for *Eco*RI and *Pst*I. The digested fragment was ligated into pKSV7. The pKSV7 with the 1000 bp fragment was used to perform allelic exchange according to [98]. Nonhemolytic colonies were identified on 5% blood agar plates (Becton Dickinson). The deletion of *hly* was further ensured by amplification of the chromosomal DNA with the primers used to generate this strain and a second set of primers that amplify the entire *hly* coding region. WT LO28 *L. monocytogenes* and the isogenic transposon insertion LO28 *hly::Tn917* mutant were gifts from Dr. Pascale Cossart (Pasteur Institute, Paris, France) [99]. *L. innocua* 33090 was purchased from ATCC. Bacteria were grown overnight at 37° C in brain heart infusion (BHI) (BD Biosciences). For invasion assays, overnight cultures were diluted 1/20 in BHI and grown at 37°C until OD_600_ = 0.7–0.8. Bacteria were washed three times in phosphate-buffered saline (PBS) and diluted to the indicated multiplicity of infection (MOI) in the cell culture medium without serum. The plasmids pAM401 and pET29b coding for *hly* were gifts from Dr. D. Portnoy (Jones and Portnoy 1994). The plasmid p*hly*/prfA* coding for *hly* was a gift from Dr. Svetlana A. Ermolaeva (Gamaleya Research Institute of Epidemiology and Microbiology, Moscow, Russia)[100]. The plasmid pQE-30 coding for *ply* was kindly provided by Dr. R. K. Tweten [40].

### Mammalian cell cultures

Human hepatocyte (HepG2 cells, ATCC HB-8065) and cervical epithelial (HeLa, ATCC CCL-2) cell lines were grown in minimum essential medium (MEM) (+) Earle's salts and L-glutamine (Invitrogen), supplemented with 10% heat inactivated fetal bovine serum (HI-FBS; Lonza), 0.1 mM nonessential amino acids, 1 mM sodium pyruvate, 100 U/ml penicillin, and 100 µg/ml streptomycin (Invitrogen). Mammalian cells were maintained at 37°C in 5% CO_2_ atmosphere_._ Cells were seeded in 24-well tissue culture plates and grown for 48 h (HepG2; 1×10^5^ cells/well) or 24 h (HeLa; 0.5×10^5^ cells/well) before infection.

### Coating of bacteria and polystyrene beads with LLO

Bacteria were coated with six-His-tagged toxin [49]. Briefly, 4×10^8^ bacteria were washed twice and incubated for 10 min on ice in buffer A (20 mM Hepes pH 7.5, 50 mM NaCl, 1 nM nickel chloride). Bacteria were washed and incubated in 200 µl buffer B (20 mM Hepes pH 7.5, 50 mM NaCl) for 10 min with six-His-tagged toxin. Bacteria were then washed once with buffer B and suspended in 200 µl of buffer C (20 mM Hepes pH 7.5, 150 mM NaCl) before infecting HepG2 cells. Carboxylate microspheres (Alexa 350, 1 µm diameter; Molecular Probes) were covalently coated with 5 mg/ml bovine serum albumin (BSA) following the manufacturer's instructions. LLO was noncovalently associated to the surface of the BSA coated beads using the same experimental procedure used to coat bacteria. In each experiment, we verified that host membranes were not damaged by LLO, as this could cause the entry of antibodies used to label extracellular bacteria. Following fixation, cells were labeled with an anti-tubulin antibody (Sigma) and secondary fluorescent antibodies. We observed that in our experimental conditions antibodies could not enter the cells as microtubules were not labeled.

### Gentamicin survival assay

HepG2 cells were infected with the *L. monocytogenes* strains at MOI 20, LLO-coated bacteria at MOI 20, or *L*. *innocua* and *L. innocua* p*hly*/prfA* at MOI 100. The plates were centrifuged at room temperature for 5 min and incubated for 30 or 60 min at 37°C. Cells were washed and incubated with 15 µg/ml (for bacteria grown to OD_600_ = 0.8) or 100 µg/ml (For *L. innocua* grown to OD_600_ = 0.2) gentamicin for 1 h or 30 min, respectively. When measuring the intracellular survival of *L. innocua* in comparison to *L. innocua* phly/prfA*, the bacteria were grown to OD = 0.2 because this bacterial density led to the highest secretion levels of LLO (data not shown). Cells were washed three times with PBS and lysed with 0.2% Triton X-100 in H_2_O. Serial dilutions of cell lysates were immediately performed in PBS and plated on BHI agar. The colony forming units (CFUs) were enumerated after 48 h of incubation at 37°C.

### Measurement of bacterial association and entry into host cells

HepG2 cells (or HeLa cells) were infected with bacteria at MOI 20. The plates were centrifuged for 5 min (230 x g) at room temperature and incubated for 30 min at 37°C. Cells were washed with PBS, fixed with PBS/4% paraformaldehyde (PFA) for 15 min at room temperature, and then washed with 0.1 M glycine in PBS and incubated for 1 h in blocking solution (0.1 M glycine, 10% HI-FBS in PBS, pH 7.4)Following fixation and blocking, extracellular bacteria, total bacteria and host cells were labeled as previously described [48]. To quantitate the numbers of bacteria and mammalian cells, 40 sets of images (DAPI, Alexa 488, Alexa 568, phase contrast) were automatically acquired for each experimental condition using a 20 X objective. MetaMorph imaging and analysis software was used to enumerate the total number of bacteria (Nt), extracellular bacteria (Ne), and mammalian cells (Nc) [48]. The percentage of internalization was calculated as (Nt - Ne)/Nt x 100. Bacterial association with host cells was calculated as Nt/Nc. The results were expressed relative to control (% control). For invasion assays in the presence of soluble LLO, LLO was added to the cell culture medium along with *L. monocytogenes*.

### Construction and purification of recombinant toxins

LLO variants were constructed by PCR-based site-directed mutagenesis using pET29b encoding native six-His-tagged LLO as a template. Mutagenic primers (Table S1) were used to construct LLOmL containing the substitutions K344C and I359C; LLOpL containing the substitutions G80C and S213C; and LLO Alexa 488 with the substitutions C484A and D69C. Mutations were introduced by amplifying *hly* from pET29b using *pfu* Ultra II fusion polymerase, followed by digestion of methylated template DNA by *DpnI* (Stratagene). The constructs were transformed in *E. coli* XL1-Blue and BL21(DE3). Mutations were confirmed by DNA sequence analysis at The Ohio State University Plant-Microbe Genomics Facility. Recombinant six-His-tagged LLO and PLY were purified from *E. coli* BL21(DE3) as described previously [101]. LLOmL and LLOpL were dialyzed overnight in the absence of reducing agents to allow for disulfide bond formation. Toxins were stored at −80°C in 1 M NaCL, 50 mM phosphate buffer, pH 8. To obtain LLO Alexa 488, LLOC484A/D69C was labeled by chemical coupling to Alexa 488-Maleimide (Molecular Probes) under conditions that lead to a dye to protein molar ratio >0.9, following the manufacturer's instructions. The fluorescent toxin was separated from the unconjugated dye by gel filtration chromatography. All the recombinant toxins purified in this study contain a six His tag.

### Hemolytic assays

Sheep erythrocytes (10% suspension; Lampire Biological) were diluted to 0.25% in PBS pH 7.4. Serial dilutions of toxins and erythrocytes were co-incubated in PBS at 37°C for 30 min in 96 well plates. Plates were centrifuged and the absorbance of the supernatant (A_540_) was measured in a PowerWave_x_340 spectrophotometer. Erythrocytes incubated with 0.1% Triton X-100 in PBS or PBS alone served to determine the maximum (100%) and minimum (0%) hemolytic activity, respectively. DTT alone had no effect on hemolysis (data no shown). The hemolytic activities of the bacteria were measured using a similar approach except that the indicated amounts of bacteria were added to the wells. The kinetic hemolytic assay in Fig. 5 was performed according to [102]. In this assay a decrease in absorbance reflects the lysis of erythrocytes.

### Western blotting analysis of LLO expression by the bacterial strains

Overnight cultures of *L. monocytogenes*, *L. innocua,* and *L. innocua* p*hly*/prfA* were diluted 1/20 in BHI and grown to OD_600_ = 0.8 (*L. monocytogenes*) or OD_600_ = 0.2 (*L. innocua,* and *L. innocua* p*hly*/prfA*) in BHI. Bacterial suspensions containing 2.0×10^8^ bacteria were collected and centrifuged. The supernatants were collected for protein precipitation and bacterial pellets were washed and lysed as follows. The supernatants (0.25 ml of *L. monocytogenes* and 1 ml of *L. innocua* cultures) were subjected to trichloroacetic acid (TCA) precipitation. One volume cold TCA was added to 4 volumes of supernatant and incubated 1 h on ice. Samples were centrifuged (11,000 g, 10 min, 4°C) and precipitates were washed twice with cold acetone. Bacterial and dried protein pellets were suspended in Laemmli's sample buffer. Bacterial lysates (10^7^ bacteria/well, ∼600 ng/well) and precipitates were subjected to SDS-PAGE and western blotting using rabbit anti-LLO (Abcam) and horseradish peroxidase-conjugated secondary antibodies (Cell Signaling).

### HepG2 perforation assay

HepG2 cells (0.5×10^5^) were cultured in glass bottom culture dishes (MatTek; 35 mm petri dish, 10 mm microwell) for 48 h. Cells were washed twice and incubated in the presence or absence of 1 mM CaCl_2_ in a buffer containing 150 mM NaCl, 1 mM MgCl_2_, 5 mM KCL, 20 mM Hepes, 10 mM Glucose, 4 µM ethidium homodimer, pH 7.4. Cells were placed on a temperature controlled microscope at 37°C and phase-contrast and fluorescence images were recorded every 10 s using a 100 X objective for 10 min. LLO (0.5 nM) was added and movies were recorded for an additional 28 min. Results were expressed as the average fluorescence intensity measured from at least 5 movies at each time point.

### LDH and cell viability measurements

HepG2 cells were incubated with LLO (1, 5, or 20 nM) or BSA/LLO-coated beads for 30 min at 37°C. Following incubation, supernatants were recovered from each sample and centrifuged at 500 x g at 4°C for 5 minutes to pellet any cells released into the supernatant. 10 µl of each supernatant was diluted into 40 µl of cell culture medium without serum in 96 well plates, and assayed for the presence of lactate dehydrogenase with the TOX7 *in vitro* toxicology assay kit according to the manufacturer's instructions (Sigma). To assess cell viability immediately or 24 h after LLO treatment, HepG2 cells were detached from wells with 0.25% Trypsin-EDTA. Cells were then mixed 1∶1 with 0.4% trypan blue for 3 minutes, after which viable (unstained) and nonviable (stained in blue) cells were enumerated with a hemocytometer.

### Toxin oligomerization (NativePAGE)

Erythrocyte ghost membranes (EGM) were prepared as described previously [44] and stored at 4°C in resealing buffer (10 mM phosphate buffer, 5 mM MgCl). EGM (6.75×10^8^) were incubated in PBS with 157 nM LLO on ice for 1 min and were transferred to 37°C for 5 min. When indicated, 4 mM DTT was added to reduce the disulfide bond in LLOmL and LLOpL. Samples were centrifuged at 15,000 x g for 15 min and the supernatant was removed and replaced with an equal volume of PBS. LLO oligomerization was analyzed using the NativePAGE Novex Bis-Tris Gel electrophoresis system. The samples were mixed with NativePAGE sample buffer containing 1% detergent, and run on a 4–16% Bis-Tris gel as described by the manufacturer (Invitrogen). LLO was detected by western blotting.

### Toxin oligomerization (transmission electron microscopy)

LLO solutions (750 nM) in buffer (20 mM Hepes, pH 7.0, ±2 mM DTT) were pipetted in Teflon wells as 13 µl droplets and coated with 1 µl of a 0.5 mg/ml lipid mixture containing 50 mol% cholesterol (Avanti) and 50 mol% 1,2 dioleoyl-*sn*-glycero-3-phosphocholine (Avanti) in chloroform. After incubation in a humid chamber at room temperature for 1 h, the LLO complexes were transferred to carbon support films on electron microscopy (EM) grids and negatively stained with 1% (w/v) uranyl acetate and observed with a FEI Tecnai F20 transmission electron microscope equipped with a Gatan Ultrascan 4K X4K CCD camera.

### Invasion assays using polystyrene beads, F-actin and EEA1 labeling

Cells were washed and incubated for 30 min with BSA-, or BSA/LLO-coated beads at a MOI = 20 in MEM. Cells were washed, fixed with PFA and blocked. Extracellular beads were labeled with an anti-BSA rabbit polyclonal antibody (Sigma) followed by a goat anti-rabbit secondary antibody conjugated to Alexa-568. The percentage of internalized beads was determined by fluorescence microscopy based on their unique (Alexa 350, intracellular) or dual fluorescence (Alexa 350 + Alexa 568, extracellular) and expressed as% intracellular beads ( =  intracellular beads/total beads * 100). EEA1 was labeled with a primary antibody (Santa Cruz) and a fluorescent (Alexa 488) donkey anti-goat secondary antibody in permeabilized cells. When co-labeling of the BSA beads and EEA1 was performed, a fluorescent donkey anti-rabbit secondary antibody was used to label the primary rabbit anti-BSA antibody. For F-actin labeling, cells were fixed and labeled as described in [102].

### Silencing of clathrin heavy chain in HepG2 cells

HepG2 cells were transfected in 24 well cell culture plates with specific human clathrin heavy chain siRNA (Ambion 43908824, Table S1) or with scrambled siRNA (Ambion, 4390843) (50 nM siRNA in 0.5 ml/0.5×10^5^ cell/well) using SiPort neoFx transfection reagent according to the manufacturer instructions (Ambion). After 24 h, cell culture medium was replaced and cells were further incubated for 24 h. Clathrin knock-down efficiency was verified in each experiment by western blotting using primary rabbit anti-clathrin heavy chain (Abcam) and mouse anti-α-tubulin (Sigma) antibodies.

### Cell treatment with chemical inhibitors

Cells were pre-incubated with 0.5 µg/ml cytochalasin D (Sigma) for 10 min, 33 µM nocodazole (Sigma) for 60 min, 250 µM Genistein (Sigma) for 60 min, 37 µM LY294002 (EMD chemicals) for 60 min, 1 µM wortmannin (Sigma) for 60 min, 80 or 120 µM dynasore (Sigma) for 30 min, and 10 µM chlorpromazine (Sigma) for 30 min before the addition of coated beads, and the drugs were maintained at the same concentrations in the cell culture medium until the cells were fixed.

### Transferrin uptake

HepG2 cells were washed three times in MEM and were serum starved for 2 h. Cells were incubated in MEM at 37°C with 5 µg/ml iron loaded Alexa 568 conjugated tranferrin (Molecular Probes). After 2 and 10 min of incubation, cells were transferred to ice, washed three times with cold medium and acid washed (200 mM NaCl, 50 mM MES, pH5.0) for 5 min. Four washes were performed with a cold buffer containing 150 mM NaCl, 1 mMCaCl_2_, 1 mM PBS, 5 mM KCl, 20 mM Hepes, pH 7.4. Alexa 488-conjugated transferrin was added (5 µg/ml) for 2 h at 4°C. Cells were then washed and fixed. Phase contrast and fluorescence images of the internalized (Alexa 568-Tf) and cell surface-associated (Alexa 488-Tf) fluorescent transferrin were acquired using a 40X objective. Extracellular and internalized transferrin molecules were measured by quantitative fluorescence microscopy. Briefly, images were first background corrected and the average fluorescence intensities were measured in the cells (about 1000 cells were analyzed for each experimental condition).

### Cholesterol depletion and repletion and LLO treatment with neutralizing antibodies

For cholesterol depletion, HepG2 cells were washed twice with MEM (without serum) and incubated at 37°C for 30 min with 5 mM MβCD in MEM. Cells were then washed twice and assayed as described. For cholesterol repletion, cholesterol-depleted cells were washed twice and incubated for 15 min with a solution of 5 mM cholesterol-MβCD in MEM, then washed and assayed as described.

A 1∶1∶1 mixture of three monoclonal anti-LLO neutralizing (H14-3, B8B20-3-2, and A4-8) or control anti-LLO (D21-1-4) antibodies (gift from Dr. P. Cossart, Institute Pasteur, France) was added to purified LLO or to BSA/LLO-coated beads at concentrations of 2, 10, or 20 µg/ml.

### Image acquisition

Images were acquired on a motorized inverted epi-fluorescence microscope (Axio Observer D1, Zeiss) equipped with 20 X Plan Neofluar (N.A. = 0.5), 100 X Plan Apo (N.A. = 1.4), and 40X Plan Neofluar (N.A. = 1.3) objectives, a high speed filter changer Lambda DG-4 (300 Watts Xenon Arc bulb, Sutter Instrument Company), an optical emission filter wheel Lambda 10–3 for the fluorescence imaging, and a Smart shutter that controls the illumination for phase contrast imaging (Sutter Instrument Company). The camera (back-illuminated, frame-transfer EMCCD Cascade II 512) was from Photometrics. The filter sets for fluorescence were purchased from Chroma Technology Corporation: DAPI (49000), GFP/FITC/Alexa488 (49002), Cy3/DsRed/Alexa568 (49005). The microscope was controlled by MetaMorph imaging software (Universal Imaging).

### Live cell imaging

All movies were acquired on the microscope stage of an inverted fluorescence microscope at 37°C using a 100X objective. Phase contrast and fluorescence images of HepG2 cells were acquired every 10 s for 40 min, LLO was added 10 min after starting recording. In some experiments, cells were incubated in calcium-free buffer as indicated. Movies were accelerated 198 times.

### Scanning electron microscopy

Following incubation with BSA/LLO-coated beads, cells were washed in PBS, fixed and processed as described in [23] with the following modifications: Coverslips were sputter coated with gold palladium at 17 mA for 75 sec with a Cressington 108 sputter coater. Samples were examined and photographed with a FEI Nova Nano scanning electron microscope operating at 5 Kv.

### Statistics

A minimum of three independent experiments were performed, each in duplicate, unless otherwise indicated. Data were expressed as mean ± Standard Error of the Mean (SEM). P-values were calculated using a standard two-tailed Student's t-test and determined significant if lower than 0.05. In figures, asterisks indicate a significant difference between the indicated experimental conditions (* p<0.05; ** p<0.005).

### Accession numbers

The NCBI Reference Sequence (RefSeq) accession numbers for the listeriolysin O gene and protein are as follows: *hly* (NC_003210.1), LLO ( ZP_05237132).

## Supporting Information

Figure S1 **LLO is required for efficient entry of two** ***L. monocytogenes*** **strains into HepG2 and HeLa cells.** (**A**) HepG2 and (**B**) HeLa cells were infected with WT (DP10403S or LO28) and corresponding isogenic LLO-deficient (Δ*hly or hly::Tn917*) bacteria (MOI  = 20) for 30 min at 37°C. Cells were washed, fixed, and labeled with fluorescent antibodies and DAPI. Bacterial association and entry were expressed relative to WT strains. Results were the mean ± SEM (n≥3).
(TIF)

Figure S2 ***L. monocytogenes*** **coating with recombinant LLO.** (**A**) The hemolytic activity of LLO Alexa 488 and LLO was measured in triplicate for 30 min at 37°C and pH = 7.4. A representative experiment (of 3) is presented. (**B**) LLO-deficient *L. monocytogenes* (Δ*hly,* DPL2161, *L.m*.) were coated with LLO Alexa 488 and the fluorescence intensity associated with bacteria was measured by quantitative fluorescence microscopy. Results were the average fluorescence intensity (A.F.I.) ± SEM expressed in arbitrary units (n≥3). At least 100 bacteria were analyzed in each experiment.
(TIF)

Figure S3 **LLO-coated beads do not affect HepG2 cell viability.** (**A**) HepG2 cells were incubated for 30 min at 37°C with BSA- or BSA/LLO-coated beads (MOI = 20). LDH released into the supernatant was measured immediately after treatment. As controls, we used untreated cells (control) and cells incubated with 0.2% TX-100 for 30 min. Cell viability was assessed immediately (**B**) or 24 h (**C**) after treatment by counting viable cells that excluded trypan blue. Results were the mean ± SEM (n≥3).
(TIF)

Figure S4 **LLO-coated beads form small pores in HepG2 cells.** HepG2 cells were incubated with BSA/LLO- and BSA-coated beads for 30 min at 37°C. Cells were pulse labeled with propidium iodide (100 µM) for 1 min, washed and fixed to quantify the fluorescence associated with the cells. (**A**) Representative DAPI and propidium iodide (PI) fluorescence images were acquired with a 20X objective. (**B**) Quantification of propidium iodide incorporation into the cells. Results represent the average fluorescence intensity (A.F.I.) per pixel in the cells and were the mean ± SEM (n≥3).
(TIF)

Figure S5 **PLY induces** ***L. monocytogenes*** **entry into HepG2 cells.** (**A)** Hemolytic activity of LLO and PLY measured after 30 min at 37°C, pH = 7.4. A representative experiment (of 3) is presented. (**B**) HepG2 cells were incubated for 30 min at 37°C with LLO-deficient *L. monocytogenes* incubated in coating buffer in the presence or absence of 1 µM LLO or PLY. Bacterial internalization was measured by fluorescence microscopy. Results were expressed as the mean ± SEM (n≥3).
(TIF)

Figure S6 **F-actin, dynamin, and clathrin are dispensable for the membrane repair pathway.** HepG2 cell perforation was measured by quantitative live cell fluorescence microscopy. Cells were incubated on the microscope stage at 37°C for 30 min with 20 µg/ml propidium iodide (a cell impermeant nuclear dye) in the presence or absence (Ca^2+^ free medium) of 1 mM extracellular calcium. When inhibitors were used, cells were pre-incubated at 37°C with 10 µM chlorpromazine (CPZ), 160 µM dynasore (DY) for 30 min or 0.5 µg/ml cytochalsin D (CD) for 10 min and the inhibitors were maintained throughout the duration of the experiments. Phase contrast and fluorescence images were recorded at regular time intervals using a 100X objective. LLO (1.2 nM) was added after 5 min of incubation. Results were expressed as the average fluorescence intensity (in arbitrary units) in the cells ± SEM of 5 to 10 movies for each experimental condition.
(TIF)

Table S1 **Primer and siRNA sequences used in this study.**
(RTF)

Video S1 **Phase contrast movie of HepG2 cells incubated in calcium-free medium.**
(AVI)

Video S2 **Fluorescence movie of HepG2 cells incubated in calcium-free medium.**
(AVI)

Video S3 **Phase contrast movie of HepG2 cells exposed to 0.5 nM LLO in calcium-free medium.**
(AVI)

Video S4 **Fluorescence movie of HepG2 cells exposed to 0.5 nM LLO in calcium-free medium.**
(AVI)

Video S5 **Phase contrast movie of HepG2 exposed to 0.5 nM LLO in calcium-free medium in the absence of ethidium homodimer.**
(AVI)

Video S6 **Phase contrast movie of HepG2 cells exposed to 0.5 nM LLO.**
(AVI)

Video S7 **Phase contrast movie of HepG2 cells exposed to 1.2 nM LLO.**
(AVI)

Video S8 **Phase contrast movie of HepG2 cells exposed to 0.5 nM LLO + 0.5** µ**g/ml cytochalasin D.**
(AVI)

Video S9 **Phase contrast movie of HepG2 cells exposed to 1.2 nM LLO and 250** µ**M genistein.**
(AVI)

Video S10 **Phase contrast movie of HepG2 cells exposed to 10 nM LLOpL.**
(AVI)
