# Supplementary figures and images for: The Pore-Forming Toxin Listeriolysin O Mediates a Novel Entry Pathway of L. monocytogenes into Human Hepatocytes
Source: PLoS Pathog. 2011 Nov 3;7(11):e1002356. doi: 10.1371/journal.ppat.1002356 (PMC3207921; doi:10.1371/journal.ppat.1002356)

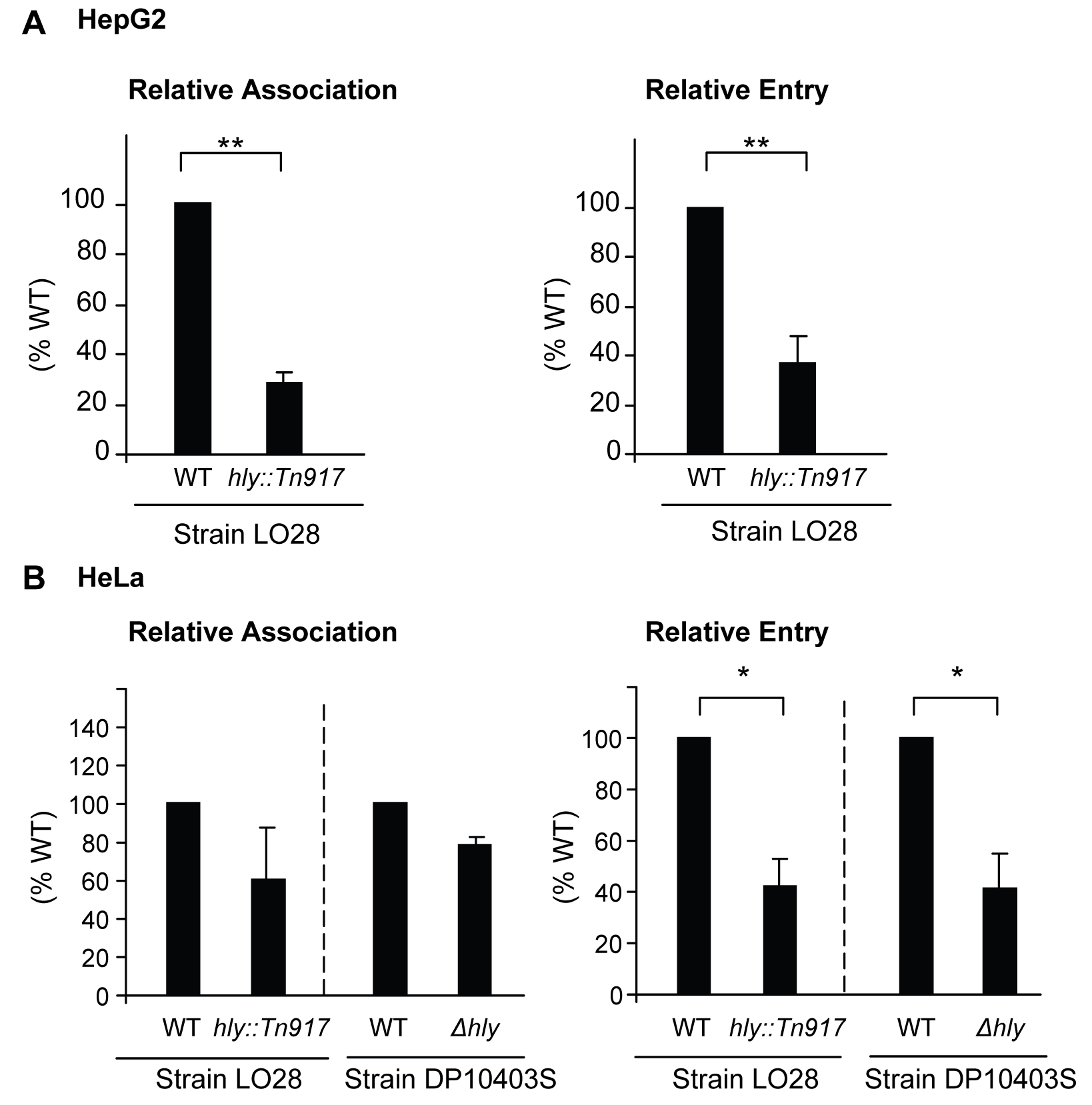

Supplement: Figure S1 — LLO is required for efficient entry of two L. monocytogenes strains into HepG2 and HeLa cells. (A) HepG2 and (B) HeLa cells were infected with WT (DP10403S or LO28) and corresponding isogenic LLO-deficient (Δhly or hly::Tn917) bacteria (MOI = 20) for 30 min at 37°C. Cells were washed, fixed, and labeled with fluorescent antibodies and DAPI. Bacterial association and entry were expressed relative to WT strains. Results were the mean ± SEM (n≥3). (TIF) [file ppat.1002356.s001.tif]

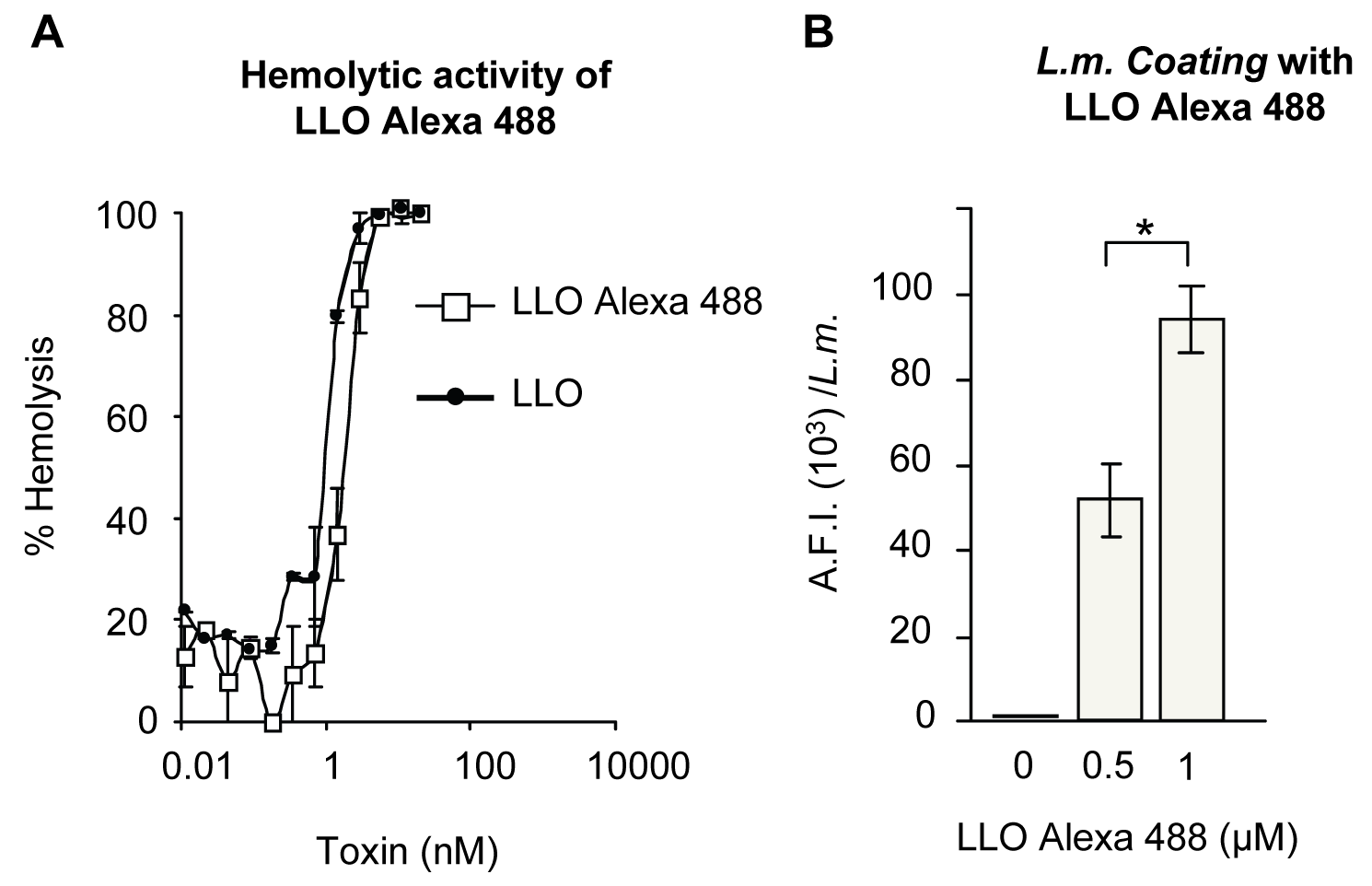

Supplement: Figure S2 — L. monocytogenes coating with recombinant LLO. (A) The hemolytic activity of LLO Alexa 488 and LLO was measured in triplicate for 30 min at 37°C and pH = 7.4. A representative experiment (of 3) is presented. (B) LLO-deficient L. monocytogenes (Δhly, DPL2161, L.m.) were coated with LLO Alexa 488 and the fluorescence intensity associated with bacteria was measured by quantitative fluorescence microscopy. Results were the average fluorescence intensity (A.F.I.) ± SEM expressed in arbitrary units (n≥3). At least 100 bacteria were analyzed in each experiment. (TIF) [file ppat.1002356.s002.tif]

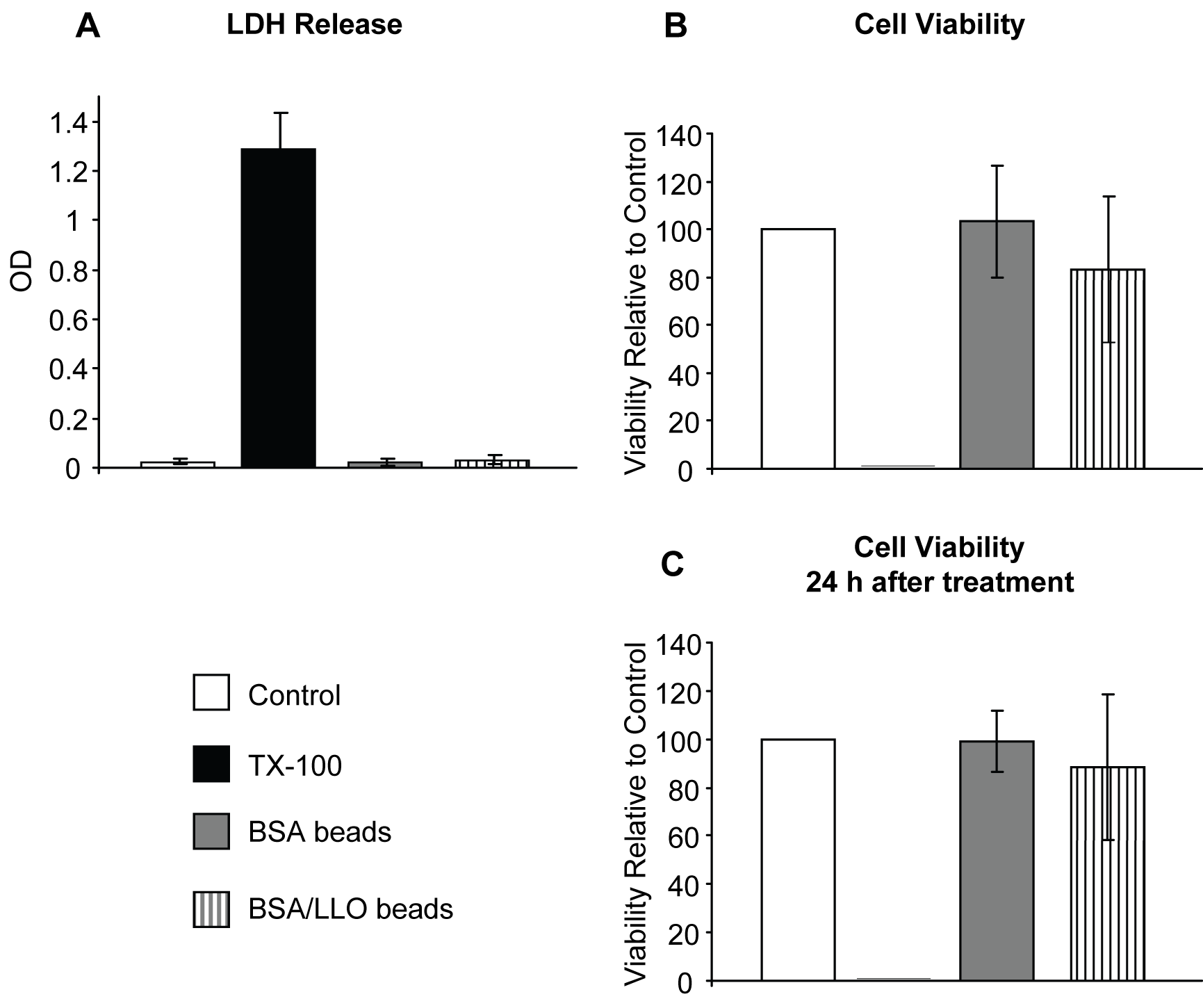

Supplement: Figure S3 — LLO-coated beads do not affect HepG2 cell viability. (A) HepG2 cells were incubated for 30 min at 37°C with BSA- or BSA/LLO-coated beads (MOI = 20). LDH released into the supernatant was measured immediately after treatment. As controls, we used untreated cells (control) and cells incubated with 0.2% TX-100 for 30 min. Cell viability was assessed immediately (B) or 24 h (C) after treatment by counting viable cells that excluded trypan blue. Results were the mean ± SEM (n≥3). (TIF) [file ppat.1002356.s003.tif]

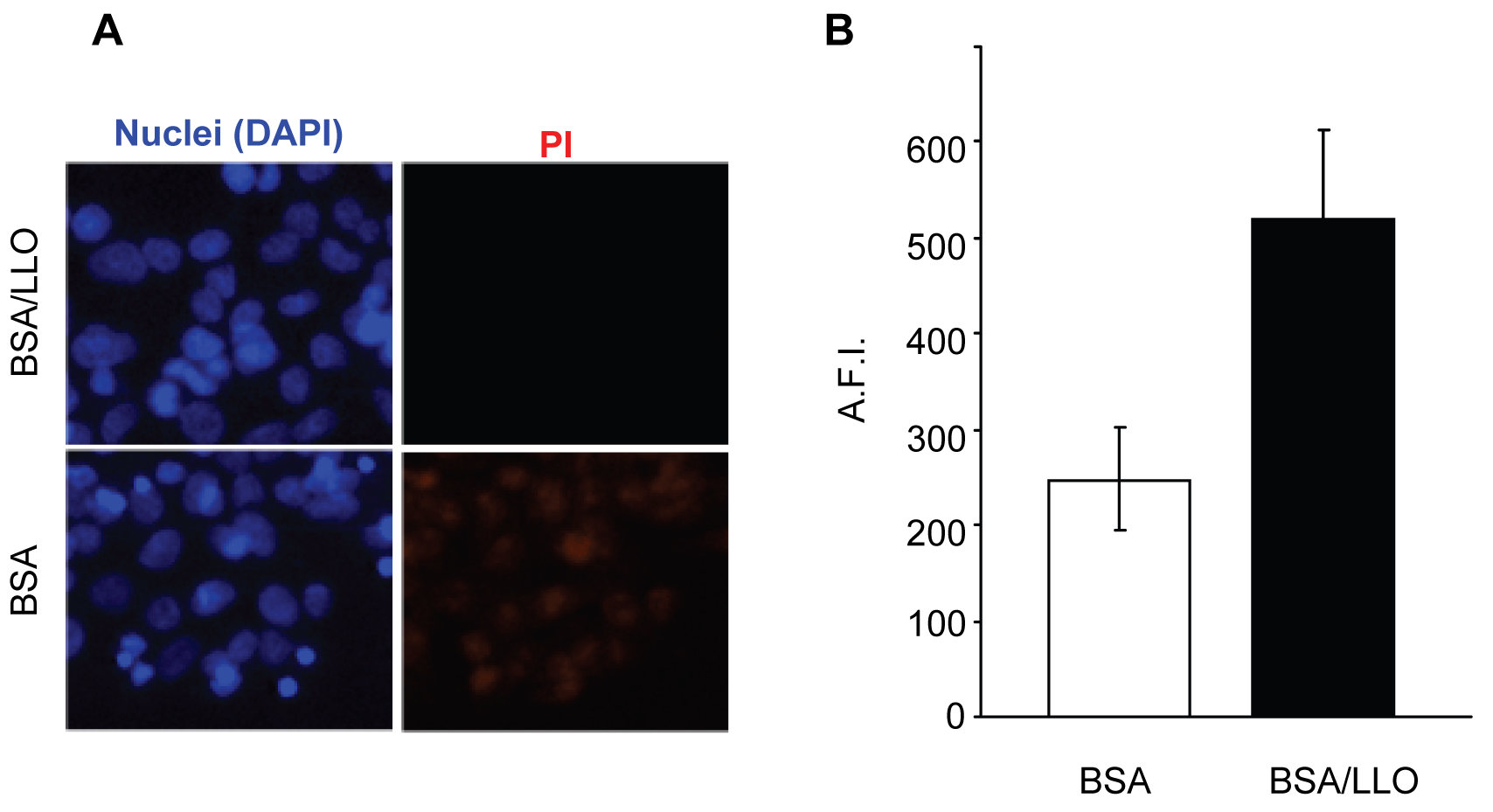

Supplement: Figure S4 — LLO-coated beads form small pores in HepG2 cells. HepG2 cells were incubated with BSA/LLO- and BSA-coated beads for 30 min at 37°C. Cells were pulse labeled with propidium iodide (100 µM) for 1 min, washed and fixed to quantify the fluorescence associated with the cells. (A) Representative DAPI and propidium iodide (PI) fluorescence images were acquired with a 20X objective. (B) Quantification of propidium iodide incorporation into the cells. Results represent the average fluorescence intensity (A.F.I.) per pixel in the cells and were the mean ± SEM (n≥3). (TIF) [file ppat.1002356.s004.tif]

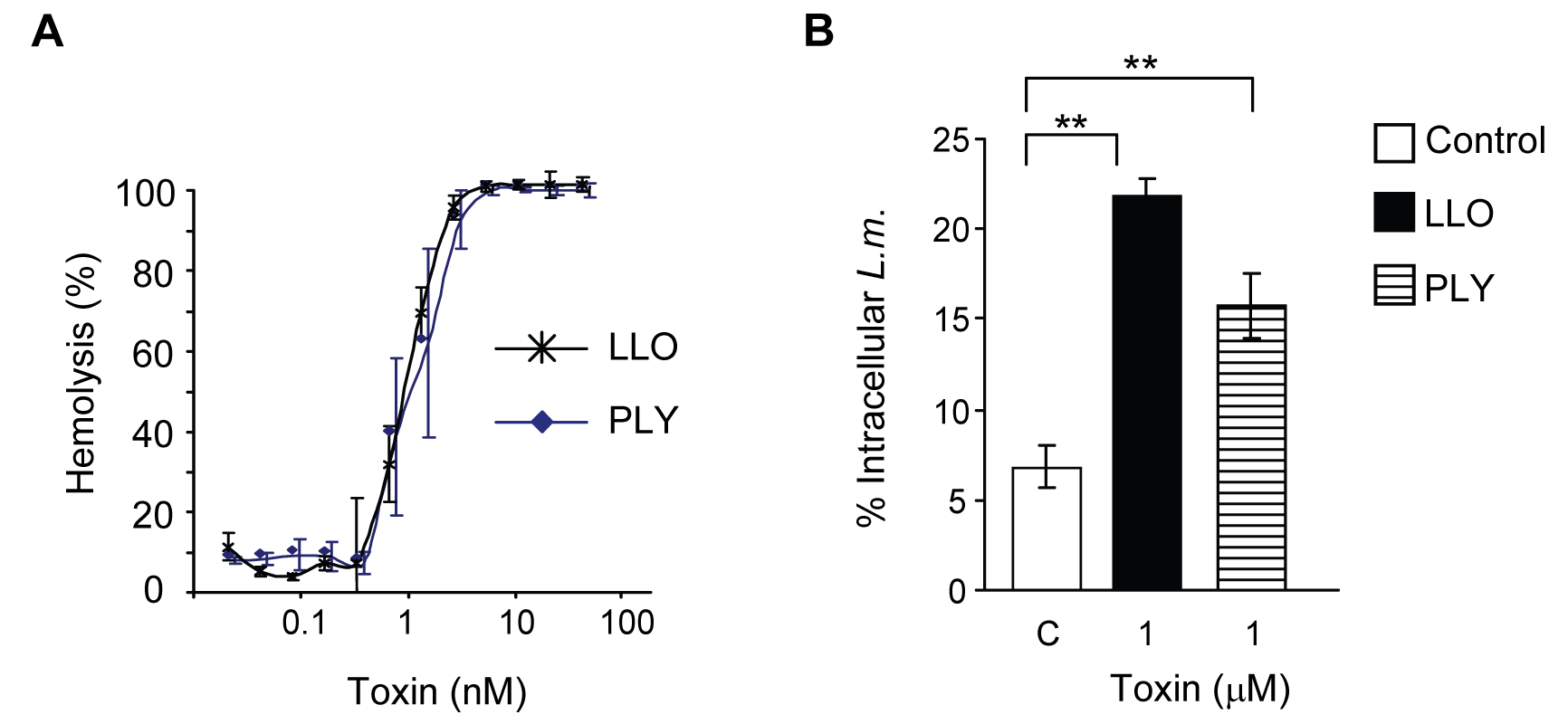

Supplement: Figure S5 — PLY induces L. monocytogenes entry into HepG2 cells. (A) Hemolytic activity of LLO and PLY measured after 30 min at 37°C, pH = 7.4. A representative experiment (of 3) is presented. (B) HepG2 cells were incubated for 30 min at 37°C with LLO-deficient L. monocytogenes incubated in coating buffer in the presence or absence of 1 µM LLO or PLY. Bacterial internalization was measured by fluorescence microscopy. Results were expressed as the mean ± SEM (n≥3). (TIF) [file ppat.1002356.s005.tif]

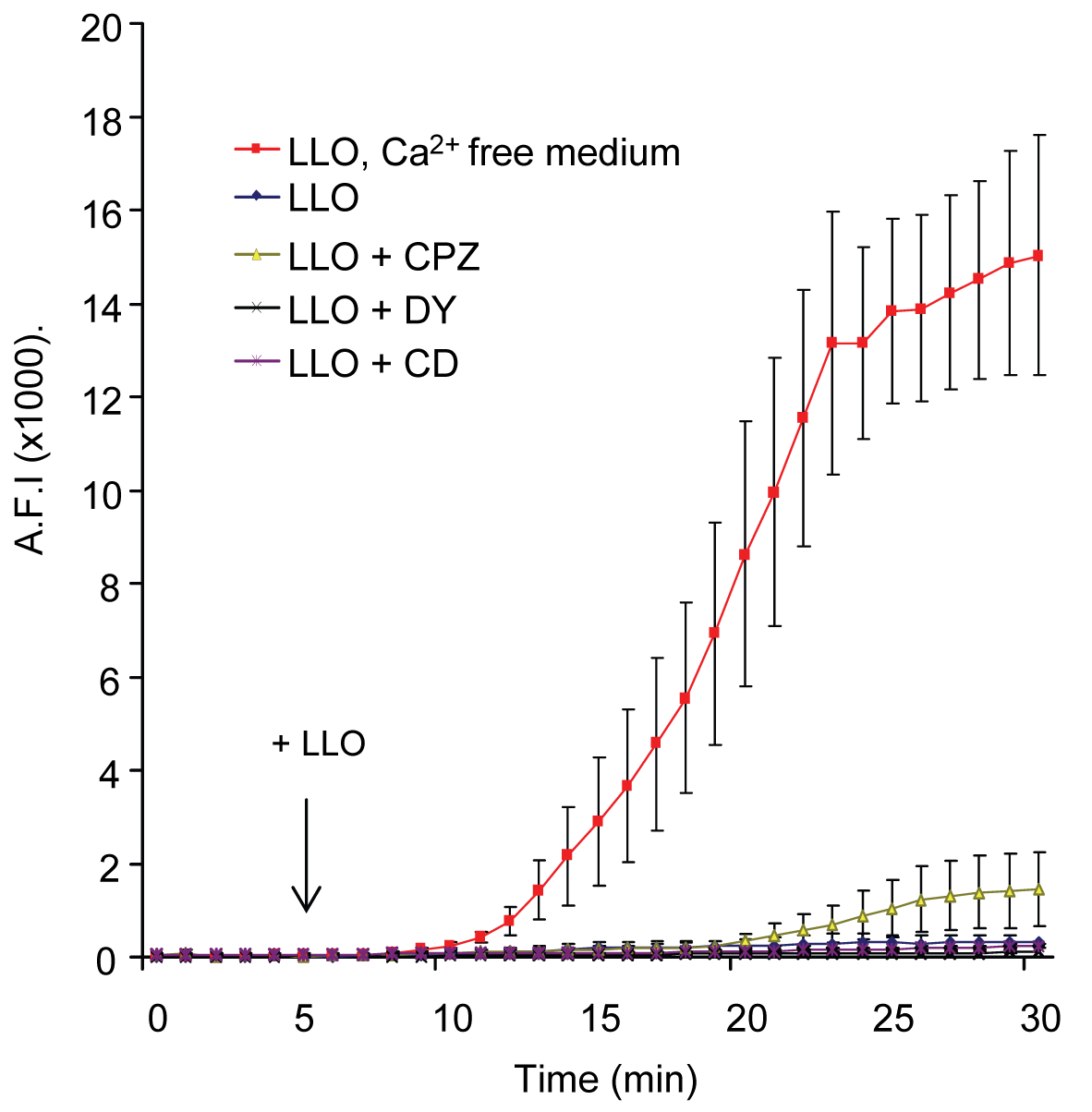

Supplement: Figure S6 — F-actin, dynamin, and clathrin are dispensable for the membrane repair pathway. HepG2 cell perforation was measured by quantitative live cell fluorescence microscopy. Cells were incubated on the microscope stage at 37°C for 30 min with 20 µg/ml propidium iodide (a cell impermeant nuclear dye) in the presence or absence (Ca2+ free medium) of 1 mM extracellular calcium. When inhibitors were used, cells were pre-incubated at 37°C with 10 µM chlorpromazine (CPZ), 160 µM dynasore (DY) for 30 min or 0.5 µg/ml cytochalsin D (CD) for 10 min and the inhibitors were maintained throughout the duration of the experiments. Phase contrast and fluorescence images were recorded at regular time intervals using a 100X objective. LLO (1.2 nM) was added after 5 min of incubation. Results were expressed as the average fluorescence intensity (in arbitrary units) in the cells ± SEM of 5 to 10 movies for each experimental condition. (TIF) [file ppat.1002356.s006.tif]
