# Supplementary material for: The Pore-Forming Toxin Listeriolysin O Mediates a Novel Entry Pathway of L. monocytogenes into Human Hepatocytes
Source: PLoS Pathog. 2011 Nov 3;7(11):e1002356. doi: 10.1371/journal.ppat.1002356 (PMC3207921; doi:10.1371/journal.ppat.1002356)
Supplement: Table S1 — Primer and siRNA sequences used in this study. (RTF) [file ppat.1002356.s007.rtf]

Table S1. Primer and siRNA sequences used in this study.
Constructs	Amino acid substitution	5' to 3' Sequences	
LLOmL	K344C	CATCAAAAATTCTTCCTTCTGCGCCGTAATACGGAGGTTCCGC	
	I359C	GATGAAGTTCAAATCTGCGACGGCAACCTCGGAGAC	
LLOpL	G80C	AATGTATTAGTATACCACTGCGATGCAGTGACAAATGTCCGCCA	
	S213C	GATGACGAAATGGCTTACTGCGAATCACAATTAATTGCG	
LLO 
Alexa 488	C484A	ATTAATGTTTACGCTAAAGAAGCGACTGGTTTAGCTTGGGAATGG	
	D69C	GAAATCGATAAGTATATACAAGGATTGTGTTATAAAAACAATGTATTAGTATACCACG	
Primers for construction of DPL2161Ähly [96]	-	GGGAATTCAATTGTTGATACAATGACATC-forward-
GGCTGCAGGGTCTTTTTGGCTTGTGTAT-reverse-	
Primers to amplify the hly ORF	-	CCGTCGGATCCATGAAAAAAATAATGCTAGTTTTTATTACAC-forward-
ATCCGCGCTGCAGTTCGATTGGATTATCTACTTTATTAC-reverse-	
Human clathrin heavy chain siRNA	-	GGUUGCUCUUGUUACGGAUtt –sense-
AUCCGUAACAAGAGCAACCgt-antisense-
	
